# Enhanced Fuel Cell Performance with Robust Pyridinium-Derivative-Functionalized SBS Triblock Copolymer Anion-Exchange Membranes

**DOI:** 10.1021/acsami.5c06877

**Published:** 2026-01-20

**Authors:** Beyadgalem Endawoke Anley, Yohannis Wondwosen Ahmed, Afandi Yusuf, Andy Candra, Sintayehu Leshe Kitaw, Tsung-Yun Wu, Chun-Chiang Huang, Jun-Sheng Wang, Darieo Thankachan, Mahvash Hira Khan, Yu-Ting Cheng, Chen-Hao Wang, Hsieh-Chih Tsai

**Affiliations:** † Graduate Institutes of Applied Science and Technology, 34878 National Taiwan University of Science and Technology, Taipei 106, Taiwan; ‡ Advanced Membrane Materials Center, 34878 National Taiwan University of Science and Technology, Taipei 106, Taiwan; § Department of Material Science and Engineering, 34878 National Taiwan University of Science and Technology, Taipei 106, Taiwan; ∥ Taiwan Instrument Research Institute, National Applied Research Laboratories, Hsinchu 302, Taiwan; ⊥ R&D Center for Membrane Technology, Chung Yuan Christian University, Chungli, Taoyuan 320, Taiwan

**Keywords:** polystyrene-*block*-polybutadiene-*block*-polystyrene, anion-exchange membranes, fuel cell, ionic conductivity, dimensional stability

## Abstract

A series of polystyrene-*block*-polybutadiene-*block*-polystyrene (SBS) membranes functionalized with pyridinium derivatives (SBS-QA^+^py) were synthesized through free-radical chlorination of the polybutadiene segments using azobis­(isobutyronitrile) (AIBN) as the free-radical initiator, promoting uniform chain growth. The resulting SBS-QA^+^py anion-exchange membranes (AEMs) were quaternized via a conventional solution-casting method. The membranes exhibited controlled ion-exchange capacities (IECs), water uptake (WU), and optimized interionic separation, achieving a balance between hydration, dimensional stability, and mechanical integrity. Noncovalent stacking interactions between the polystyrene and pyridinic segments significantly contributed to these properties. Notably, the SBS-Qdpy_2_ AEM achieved a peak ionic conductivity of 101.23 mS cm^–1^ at 80 °C (IEC: 1.72 mequiv g^–1^) and a peak power density of 398.14 mW cm^–2^ in a H_2_/O_2_ flow single cell at 80 °C, surpassing the performance of previously reported SBS-based AEMs. The membranes also demonstrated excellent chemical durability in 1 M NaOH solutions over 30 days, highlighting superior alkaline stability. These results underscore the critical role of optimized ion exchange and membrane morphology enhancing the fuel cell performance, positioning SBS-QA^+^py AEMs as promising candidates for next-generation fuel cells. Optimizing grafting, quaternization, and cross-linking will create stable AEMs with selective, well-defined nanoionic channels for efficient anion diffusion.

## Graphical abstract

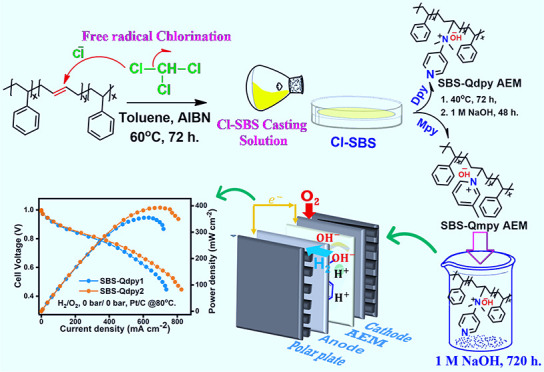

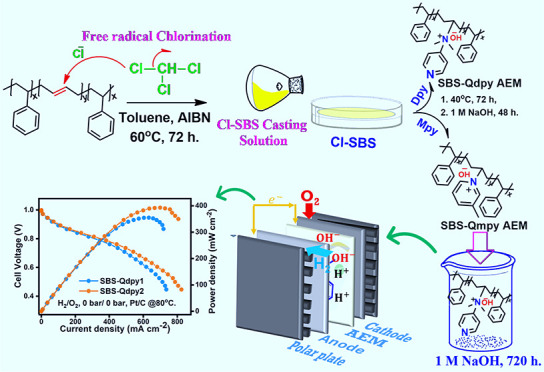

## 1 Introduction

Due to growing energy demands driven by population growth, sustainable energy conversion systems are essential to securing future energy supplies. These energy demands are closely related to societal well-being and economic resilience.^1,2^ Ensuring access to affordable and reliable energy is essential, especially because climate change mitigation requires emissions regulation and the promotion of renewable energy conversion alternatives.^3,4^ The depletion of nonrenewable resources and associated environmental impacts make green energy an urgent global priority.^5^ The intermittency of these renewable sources, such as solar and wind, highlights the need for advances in electrochemical energy conversion and storage technologies.^6^ Clean energy-converting devices, which convert green fuels to electricity with environmentally benign byproducts like water, are critical to the advancement of future sustainability.^7,8^ Here, green hydrogen, recognized for its sustainability and high energy content, is a promising energy carrier in anion-exchange membranes (AEMs).^9^ AEM serves as a core component in electrochemical cells such as fuel cells, water electrolyzers, and flow batteries, functioning as both physical separators and selective ion conductors.^10^ Anion-exchange-membrane fuel cells (AEMFCs) are promising, cost-effective alternatives to proton-exchange-membrane fuel cells because they enable the use of non-noble-metal catalysts and offer enhanced kinetics for oxygen reduction and hydrogen evolution reactions in alkaline environments.^11,12^ However, their widespread adoption hinges on developing AEMs with high ionic conductivity, robust mechanical strength, and long-term chemical and dimensional stability.^13^ Achieving this delicate balance remains challenging due to persistent issues such as excessive swelling at high ion-exchange capacity (IEC), cationic site degradation under alkaline conditions, phase separation instability, and poor compatibility between hydrophilic and hydrophobic domains.^14^ Furthermore, the trade-off between conductivity and mechanical durability and a limited understanding of ion-transport mechanisms at the molecular level continue to hinder the rational design of high-performance AEMs. These challenges underscore the need for innovative molecular architectures, durable cationic headgroups, and structurally reinforced polymer backbones that are capable of withstanding harsh electrochemical environments.

Poly­(styrene-*block*-butadiene-*block*-styrene) (SBS), a widely used thermoplastic elastomer, has recently gained attention as a polymer backbone for AEMs due to its exceptional mechanical strength, processability, and chemical resistance.^15^ Notwithstanding, the brilliant properties of SBS are restricted, just being applied in tires, shoe wear, compatibilizers, and so forth. There is a need to investigate the expected utilization of SBS in smart polymeric regions. SBS consisted of one soft block made of polybutadiene (PB) and two hard blocks made of polystyrene (PS). These blocks had the ability to self-assemble into highly organized microdomain-separated phase structures with molecular-scale dimensions, with the PS segment morphology serving as physical cross-links.^16^ Recently, the SBS triblock copolymer has emerged as a promising backbone for AEMs due to the remarkable stability imparted by its aryl ether linkages, which are inherently resistant to chemical degradation;^17,18^ it has remarkable mechanical properties and enhanced ionic conductivity; its low dielectric effect, stemming from its unique triblock structure, further contributes to its suitability.^19^ Recently, a series of side-chain-type AEMs were designed via free-radical-type grafting of SBS; these membranes exhibited remarkable conductivity and alkaline stability, showcasing their potential for advanced applications.^20^ Its unique ether-bond-free backbone and triblock structure make SBS a promising material for AEM applications. SBS stands out as a thermoplastic elastomer that demonstrates commendable resistance to both acidic and basic environments while being soluble in common organic solvents, such as toluene, tetrahydrofuran, and chloroform. These attributes make SBS a promising candidate as a base polymer for AEMs, offering potential benefits such as reduced costs, elasticity, and a recyclable nature.^20^ However, unmodified SBS lacks hydrophilicity and does not provide inherent ion-transport channels, severely limiting its ionic conductivity. Additionally, AEMs face issues such as cation erosion, dissolution, chemical instability, and mechanical degradation due to hydroxide ion attacks on the cationic sites. Functionalizing SBS with pyridinium derivatives offers a promising strategy to address these challenges by introducing well-defined ionic domains, followed by subsequent chlorination of the PB segments. Moreover, the opportunistic π–π noncovalent interactions between the pyridine rings and the PS blocks promote microphase compatibility and structural integrity beyond conventional covalent bonding. This not only facilitates stable ion-conducting pathways but also enhances the membrane’s chemical and mechanical robustness.^21^ The stacking system creates steric hindrance, shielding the cationic sites and reducing vulnerability to hydroxide ion attacks, thereby improving its overall stability and mitigating cation erosion in AEMs.^22^ These findings highlight the potential of SBS–pyridine derivatives and underscore the need for further optimizations to enhance the physicochemical properties.

In this study, we functionalized SBS through direct free-radical chlorination of the PB segments, followed by quaternization with pyridine derivatives. This approach offers an alternative to hydrogenation and epoxidation of SBS, which often rely on toxic and costly catalysts, particularly for SEBS-based AEMs. This method leverages the reactivity of the double bonds in SBS, facilitating uniform polymer chain growth and a tailored polymer architecture. The resulting SBS-based AEMs exhibited enhanced ion-transport efficiency, robust mechanical properties, and minimal swelling, even under high temperatures, due to the elastomeric nature of SBS. These properties make the functionalized SBS a promising material for AEMs, offering cost-effectiveness, elasticity, good physical separation between PS and PB segments, and recyclability. Furthermore, the pyridinium groups provide additional stabilization through resonance effects and π–π stacking, collectively reinforcing ion-transport channels and improving alkaline durability. These results demonstrate how targeted chemical modification coupled with tailored noncovalent interactions can significantly elevate the membrane performance, offering a structurally tunable platform for advanced ion-exchange materials.

## 2 Experimental Section

### 2.1 Materials

Polystyrene-*b*-polybutadiene-*b*-polystyrene (98%, Sigma-Aldrich, 30% polystyrene, average MW of 140 kDa) was purchased from Sigma-Aldrich and purified using methanol from a chloroform solution, anhydrous chloroform (98%, Sigma-Aldrich), dimethylacetamide (99%, Sigma-Aldrich), and methanol (99.8%, Honeywell), dried by a molecular sieve. Azobis­(isobutyronitrile) (AIBN, 99%) was purified from methanol by recrystallization, 2,6-di-*tert*-butyl-*p*-hydroxytoluene (BHT, 99.0%, Sigma-Aldrich) was used as a free-radical inhibitor, and anhydrous toluene (99.7%, Sigma-Aldrich) was used as a solvent. 4-(Dimethylamino)­pyridine (99%, Aelfa-Asar) and 3-methylpyridine [39% (w/v), Sigma-Aldrich] were used as quaternization agents after being dried by a molecular sieve. A platinum–carbon (60% Pt/C) catalyst and isopropyl alcohol (IPA, 98%) were employed to prepare the catalyst ink. Deionized water was used in the laboratory from our laboratory dispenser for all experiments; all reagents and solvents used were of analytical grade.

### 2.2 Synthesis of a Chlorinated SBS (CSBS) Tricopolymer

The synthesis was carried out in a two-necked reactor equipped with a mechanical stirrer and a nitrogen inlet, following a conceptual framework adapted from related studies (Schemes 1 and 2).^20,23^ Methanol-purified SBS (2.0 g, 37 mmol) was dissolved in anhydrous toluene (60 mL) at 50 °C under stirring until a homogeneous solution was obtained. After the mixture was cooled to room temperature, a 1 wt % AIBN solution in anhydrous chloroform (2 mL) was introduced under N_2_ flow, followed by the dropwise addition of anhydrous chloroform [13.26 mL, 111 mmol; chloroform/polymer = 1:3 (w/w)]. The reaction mixture was maintained at 60 °C for 72 h under N_2_ at 150 rpm. As the viscosity increased, the stirring speed was reduced to ensure uniform mixing. The reaction was terminated by cooling to room temperature and adding BHT (4 g in 10 mL of toluene) to quench residual radicals. The product was filtered, precipitated in excess methanol, and washed repeatedly with methanol. The precipitate was redissolved in chloroform, reprecipitated in methanol, and dried under vacuum to constant weight. The obtained CSBS was solution-cast into membranes and subsequently quaternized with pyridinium derivatives of 4-(dimethylamino)­pyridine (dpy) and 3-methylpyridine (mpy) at loadings of 10 and 20 wt % to yield SBS-QA^+^py AEMs (where A^+^ denotes the pyridinium cation). The successful formation of copolymers was confirmed by ^1^H NMR, FT-IR (ATR), and XPS analyses.

**Scheme 1:**
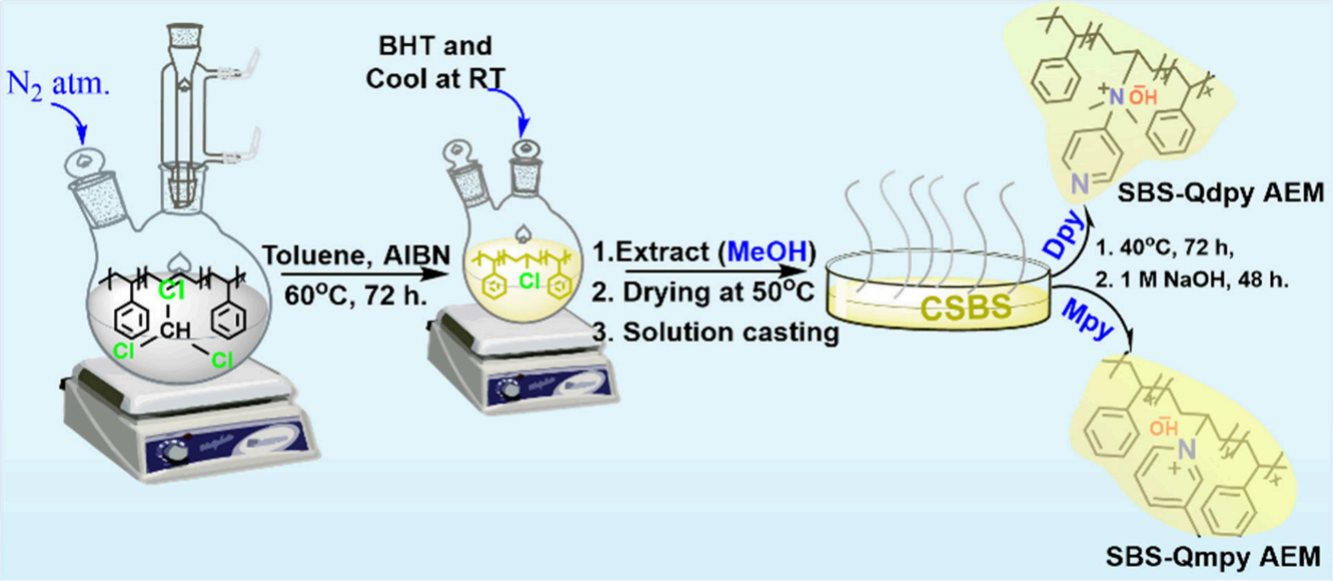
Schematic Flow Chart Illustrating the Chlorination of SBS and Quaternization Processes

**Scheme 2:**
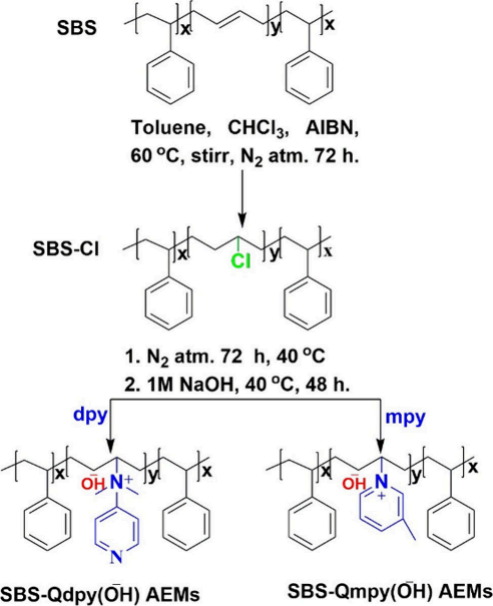
Synthesis Routes for Free-Radical Chlorination of SBS and Pyridine-Derivative-Based Quaternizations

### 2.3 Preparations of SBS-QA^+^py Membranes

The solution-casting method was employed to prepare quaternized membranes of CSBS using pyridine derivatives of 4-(dimethylamino)­pyridine (Qdpy) and 3-methylpyridine (Qmpy) as quaternizing agents at loading ratios of 10% and 20%. Briefly, CSBS (10%) was dissolved in chloroform and cast onto a Teflon plate. This membrane was then dried under vacuum at 50 °C for 24 h to achieve a constant weight, followed by evaporation of the solvent at room temperature. Following this, the CSBS membrane was immersed in 10% aqueous solutions of the pyridine derivatives at a controlled temperature of 40 °C for 72 h to prepare Qdpy_1_. Similarly, 10% and 20% solutions of Qdpy_2_, Qmpy_1_, and Qmpy_2_ were prepared in the same way. This step allowed the pyridine derivatives to react with the chlorinated sites on the SBS membrane, forming the quaternized SBS-QA^+^py membranes with approximate thicknesses of 45–50 μm. After the quaternization reaction, the membranes were thoroughly washed with deionized water to remove any unreacted pyridine derivatives and other byproducts, followed by drying under vacuum. Furthermore, the membranes were immersed in a 1 M NaOH solution at room temperature for 48 h to convert them to the hydroxide form. Finally, the hydroxide forms SBS-QA^+^py membranes, which were conditioned in degassed distilled water for further characterization.

### 2.4 Instrument and Characterization

#### 2.4.1 Characterization of SBS-QA^+^py Membranes

^1^H nuclear magnetic resonance [^1^H NMR; Agilent 600 NMR system (600 MHz)] was used to confirm the chemical compositions of SBS-QA^+^py AEMs using CDCl_3_ as the solvent and tetramethylsilane as the internal standard. Attenuated-total-reflectance Fourier transform Infrared (ATR-FT-IR) spectroscopy (Bruker Scientific) was conducted with a scan rate of 32 s across a spectral range of 600–4000 cm^–1^ to identify the functional groups and molecular structure of the membrane. Gel permeation chromatography (GPC) was used to determine polymerization progress and molecular mass evolution. Thermogravimetric analysis (TGA8000, PerkinElmer) and differential scanning calorimetry (DSC8000, PerkinElmer) were used to analyze the thermal stability and thermal transitions. The mechanical properties were tested using a universal testing machine (MTS, E44.104), and X-ray photoelectron spectroscopy (XPS; Thermo-Scientific K-Alpha) was employed to determine the surface chemistry. Field-emission scanning electron microscopy (FESEM; JEOL JSM-6900LV) and atomic force microscopy (AFM; Bruker Nanoscope V) in tapping mode were employed for morphological and microstructural analyses to examine the surface topography of the membranes. The water contact angle (WCA) was measured using a Data Physics OCA 25 to determine the hydrophilicity or hydrophobicity of the membrane surface using a four-probe electrode method. Electrochemical impedance spectroscopy (Solatron Analytical 1287, R Ω) was used to measure the impedance of SBS-QA^+^py AEMs, and an electrochemical workstation (A Bio-Logic VSP300) was employed to test the H_2_/O_2_ flow performance of SBS-Qdpy_1_ and SBS-Qdpy_2_ AEMs.

#### 2.4.2 Water Uptake (WU), Swelling Ratio (SR), Hydration Number (λ), IEC, and Surface Properties

The WU and SR of the OH^–^-form AEMs were evaluated from the weight and dimensional changes of dry and hydrated membranes. Membranes (predried under vacuum at 60 °C for 12 h) were immersed in deionized water at 20, 40, 60, and 80 °C for 24 h, and the WU and SR were calculated using eqs 1 and 2:

1 $$\mathrm{WU}=\frac{M_{\mathrm{wet}}-M_{\mathrm{dry}}}{M_{\mathrm{dry}}}\times 100\%$$

2 $$\mathrm{SR}=\frac{A_{\mathrm{wet}}-A_{\mathrm{dry}}}{A_{\mathrm{dry}}}\times 100\%$$

where *M*_wet_ and *M*_dry_ are the masses of hydrated and dry membranes, respectively, and *A*_wet_ and *A*_dry_ denote the corresponding surface areas. λ, representing the number of water molecules per ionic site, was derived from eq 3:

3 $$\lambda =\frac{10\times \mathrm{WU}}{\mathrm{IEC}\times 18}$$

IEC (mequiv g^–1^) was determined via acid–base back-titration, where OH^–^-form membranes were equilibrated in 0.1 M HCl for 24 h and the released protons were titrated with 0.1 M NaOH using phenolphthalein as an indicator. IEC was calculated using eq 4:

4 $$\mathrm{IEC}=\frac{C\left(V_{1}-V_{2}\right)}{m_{\mathrm{dry}}}$$

where *C* is the NaOH concentration, *V*_1_ and *V*_2_ are the volumes of HCl and NaOH, respectively, and *m*_dry_ is the dry membrane mass. Theoretically, the IEC values were further validated by integrating the peak areas of the functional group in the ^1^H NMR spectra, as per the internal reference standard. The surface hydrophilicity was evaluated by WCA measurements using the sessile drop method (10 μL droplet, 0.5 μL s^–1^) on a Data Physics OCA 25 instrument, and the reported values represent the average of three replicates.

#### 2.4.3 Thermomechanical Properties of the Membranes

The thermal stability of the membrane was measured using thermogravimetric analysis (TGA8000, PerkinElmer) with a temperature range of 20–800 °C at a heating rate of 10 °C min^–1^ under a N_2_ atmosphere. Before testing, the membrane samples were all dried under vacuum at 80 °C for 24 h to remove the solvent residue. The mechanical properties of AEMs were measured by using a universal testing machine (MTS, E44.104) at a rate of 5 mm min^–1^ at room temperature in the dry state.

#### 2.4.4 Membrane Morphology and Computational Insights via Density Functional Theory (DFT)

The surface and cross-sectional morphologies of SBS-QA^+^py AEMs were characterized by FESEM (JEOL JSM-6900LV), while tapping-mode AFM (Bruker Nanoscope V) provided insight into nanoscale hydrophilic–hydrophobic phase separations. Small-angle X-ray scattering (SAXS) measurements (Xuess 2.0, λ = 1.54 Å) were conducted on vacuum-dried samples (70 °C, 48 h) to determine interionic domain spacing using the relations

5 $$q=\frac{4\pi \;\sin\theta }{\lambda_{\max}}$$

and

6 $$d=\frac{2\pi }{q}$$

where θ is the scattering angle and *q* is the scattering vector according to Bragg’s law of diffraction. The interdomain distance of the membranes was calculated using eqs 5 and 6, respectively. The surface chemistry and binding energies were analyzed via XPS (Thermo-Scientific K-Alpha, Al Kα source), calibrated to the C 1s signal. Complementary DFT calculations were performed using *Gaussian 09* with the B3LYP-D3 functional and 6-311++G­(d,2p) basis set, incorporating the polarizable continuum model solvation to optimize the ground-state geometries of SBS-Qdpy and SBS-Qmpy AEMs.^24,25^ These computations elucidated the lowest unoccupied molecular orbital (LUMO) energies, molecular electrostatic potentials, and hydrophilic–hydrophobic domains, thereby supporting the experimental values.

#### 2.4.5 Ionic Conductivity (σ) and Activation Energy (*E*_a_) Measurement

The ionic conductivities of both the OH^–^and Cl^–^ forms of the SBS-QA^+^py AEMs (2 × 2 cm^2^) were measured using a four-probe alternaing-current impedance method (Solartron Analytical 1287, RΩ) with an amplitude of 50 mV. To measure the OH^–^ conductivity, the membranes were first equilibrated in 1 M NaOH for 24 h, thoroughly rinsed with deionized water, and immediately tested, followed by sweeping with tissue paper. Furthermore, the Cl^–^ conductivity was determined after equilibration in deionized water to ensure full hydration, and all measurements were conducted between 20 and 80 °C in 20 °C increments. The ionic conductivities (mS cm^–1^) of OH^–^ and Cl^–^ were calculated using eq 7.

7 $$\delta \;\left({\mathrm{mS cm}}^{-1}\right)=\frac{\mathrm{thickness of the membrane}\;\left(L\right)}{\mathrm{membrane resistance}\;\left(R\right)\times \mathrm{surface area}\;\left(A\right)}$$

Moreover, the conductivity of the sample was measured after the membrane was fully immersed in deionized water for 24 h. To remove excess salt impurities, the observed activation energy (*E*_a_) of the SBS-QA^+^py series of AEM group membranes was also calculated using the Arrhenius equation of *E*_a_ = −*bR*, where −*b* is the slope of ln σ versus 100*T* ^–1^ plots and *R* is the ideal gas constant (8.314 J mol^–1^ K^–1^) from the linear functions of *k* = *A* exp­(−*E*_a_/*RT*).

#### 2.4.6 Oxidative and Alkaline Stability

The oxidative stability of OH^–^-form AEMs was evaluated by immersion in a 3% Fenton’s reagent (30% H_2_O_2_ containing 2 ppm of FeSO_4_) at 25 °C, with the residual mass recorded every 24 h over 240 h. Additionally, the alkaline stability was evaluated by aging the membranes in 1 M NaOH at 80 °C, and the residual ionic conductivity was monitored for 720 h at 80 °C with periodic rinsing in distilled water. For all measurements, the reagent solutions were refreshed at each analysis step to ensure consistency.

#### 2.4.7 Fuel Cell Performance Testing

The performance of the H_2_/O_2_ single cell was measured on a Bio-Logic VSP300 station using membrane electrode assemblies (MEAs) prepared by the catalyst-coated membrane method. A catalyst ink was prepared from 30 mg of 40% Pt/C (0.4 mg cm^–2^ loading), 2.5 g of IPA, and 150 mg of a 5 wt % SBS-Qdpy ionomer solution (20 wt % ionomer), with Pt/C prewetted to enhance dispersion and ionomer adsorption. The homogeneous suspension was applied to a 4 cm^2^ OH^–^-form membrane (45–50 μm), followed by hydration for 12 h. Then the membrane was hot-pressed between gas diffusion layers. Polarization curves were measured under fully humidified H_2_ and O_2_ (1000 mL min^–1^) of the anode and cathode at 60 and 80 °C, and the durability was evaluated at a current density of 160 mA cm^–2^ for 120 h under a steady state.

## 3 Results and Discussion

### 3.1 Synthesis and Structural Evolution

Comb-shaped anion-conductive membranes were synthesized from SBS via PB segment chlorination, followed by subsequent quaternization, generating stable pyridine-derivative-derived ionic channels essential for efficient ion transport. As shown in Figure 1, the primary step in the functionalization of SBS involves the chlorination of a PB segment, referred to as CSBS (Schemes 1 and 2).^26^ The ^1^H NMR spectrum of the block copolymer exhibits a signal at 3.98 ppm (H_12_), corresponding to the CH–Cl methylene protons on chlorination. Integration of this peak relative to the PB vinylic protons (5.0–5.5 ppm) indicates nearly quantitative chlorination of the PB segments, and the aromatic protons of the PS blocks (6.30–7.30 ppm) remained invariant throughout the functionalization. These were used as an internal reference for all quantitative analyses.^27^ In contrast, the symmetric pyridine-derived primary and aromatic protons (H_18–21_) appeared between 8.21 and 6.46 ppm including the PS segment, verifying successful quaternization of the chlorinated PB segment. The characteristic peaks at 3.69 and 3.00 ppm, assigned to the methyl protons of the quaternized amino groups, respectively, along with the integration ratios of H_12_ (3.98 ppm) relative to H_15_ (3.69 ppm) and H_17_ (3.00 ppm), are 1:1 and 3:1, respectively, closely matching the theoretical values and confirming the successful synthesis of Qdpy. Furthermore, the spectrum related to SBS-Qmpy exhibited distinct methyl and methylene resonances at 2.70 and 2.15 ppm, with quaternized aromatic peaks at 8.52–8.78 ppm, along with the integral ratios of 1:1. These results demonstrate a highly selective and well-controlled transformation along the SBS backbone, where quaternization preferentially occurs at the more nucleophilic pyridyl sites related to dpy due to electron withdrawing compared to the primary amine.^28^ The incorporation of ionic pyridinium groups enhanced chain polarity and markedly reduced the solubility in some organic solvents, reflecting strengthened interchain electrostatic interactions and the onset of polarity-driven microphase aggregation. Such organized ionic domains facilitate continuous ion-conductive pathways, which are critical for the transport of hydroxide in AEMs. Furthermore, FT-IR spectra (Figure S2) corroborate these findings, exhibiting characteristic absorptions associated with CH–Cl, pyridinium, and quaternary ammonium functionalities, affirming the successful stepwise modification of SBS.^29^

**Figure 1:**
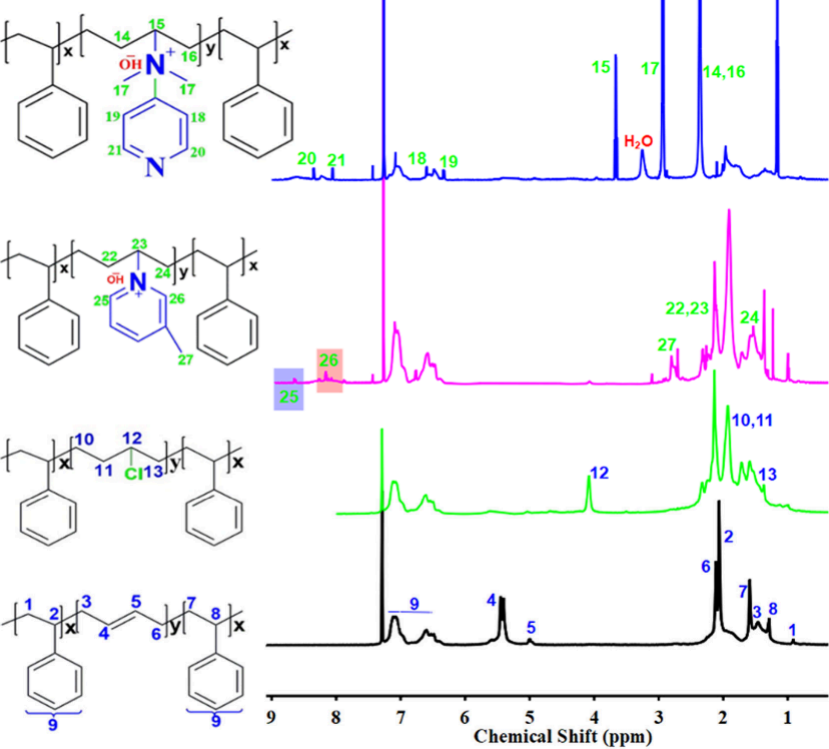
^1^H NMR spectra of SBS, CSBS, and SBS-QA^+^py AEMs in (CDCl_3_, 600 MHz).

GPC traces and ^1^H NMR analyses collectively confirm the successful incorporation of pyridinium derivatives into SBS (Figure S3). The slight polydispersity index (PDI) increase (SBS-Qmpy1, 1.137; SBS-Qmpy_2_, 1.138; SBS-Qdpy_1_, 1.140; SBS-Qdpy_2_, 1.141) reflects controlled grafting, localized chain extensions, and π–π interactions with no chain scission or cross-linking. The consistent PDI values, along with distinct ^1^H NMR shifts, indicate uniform functionalization, minimized aggregation, and stable molecular weight distribution.^30^

XPS analysis elucidated the membranes’ surface chemistry, revealing the expected binding energies, elemental composition, and local chemical environments. As shown in Figure 2a, deconvolution of the C 1s peak at ∼284 eV confirms the pristine SBS backbone through sp^2^-hybridized C=C signatures,^31^ while minor peaks at 285.6 and 288 eV indicate sp^3^ C–C/C–H environments. Further modifications are evident in Figure 2b, where Cl 2p peaks at 198 eV (Cl 2p_1/2_) and 201 eV (Cl 2p_3/2_) confirm the successful chlorination of the PB segments of SBS, a key step for pyridine derivative functionalization. Due to electronic and structural differences between dpy and mpy moieties, SBS-Qdpy_1_, SBS-Qdpy_2_, SBS-Qmpy_1_, and SBS-Qmpy_2_ exhibit distinct N 1s spectra. As shown in Figure 2c,d, deconvoluted peaks at 400 eV (SBS-Qdpy) and 399 eV (SBS-Qmpy) correspond to tertiary amine (N)­CH_3_ functionalities, while pyridinic N 1s signals at 398–399 eV are further confirmation. A peak at 401 eV, assigned to quaternary C–N^+^ bonds, confirms quaternization and the presence of ion-conducting sites. The Cl 2p spectra in Figure 2e reveal trace chloride residues postquaternization, likely due to trapped intermediates. The wide-scan XPS spectra (Figure 2f) show carbon and nitrogen as dominant elements, supporting the successful incorporation of pyridine derivatives and the intended modulation of surface chemistry consistent with stabilized nanophase separation.^32^

**Figure 2:**
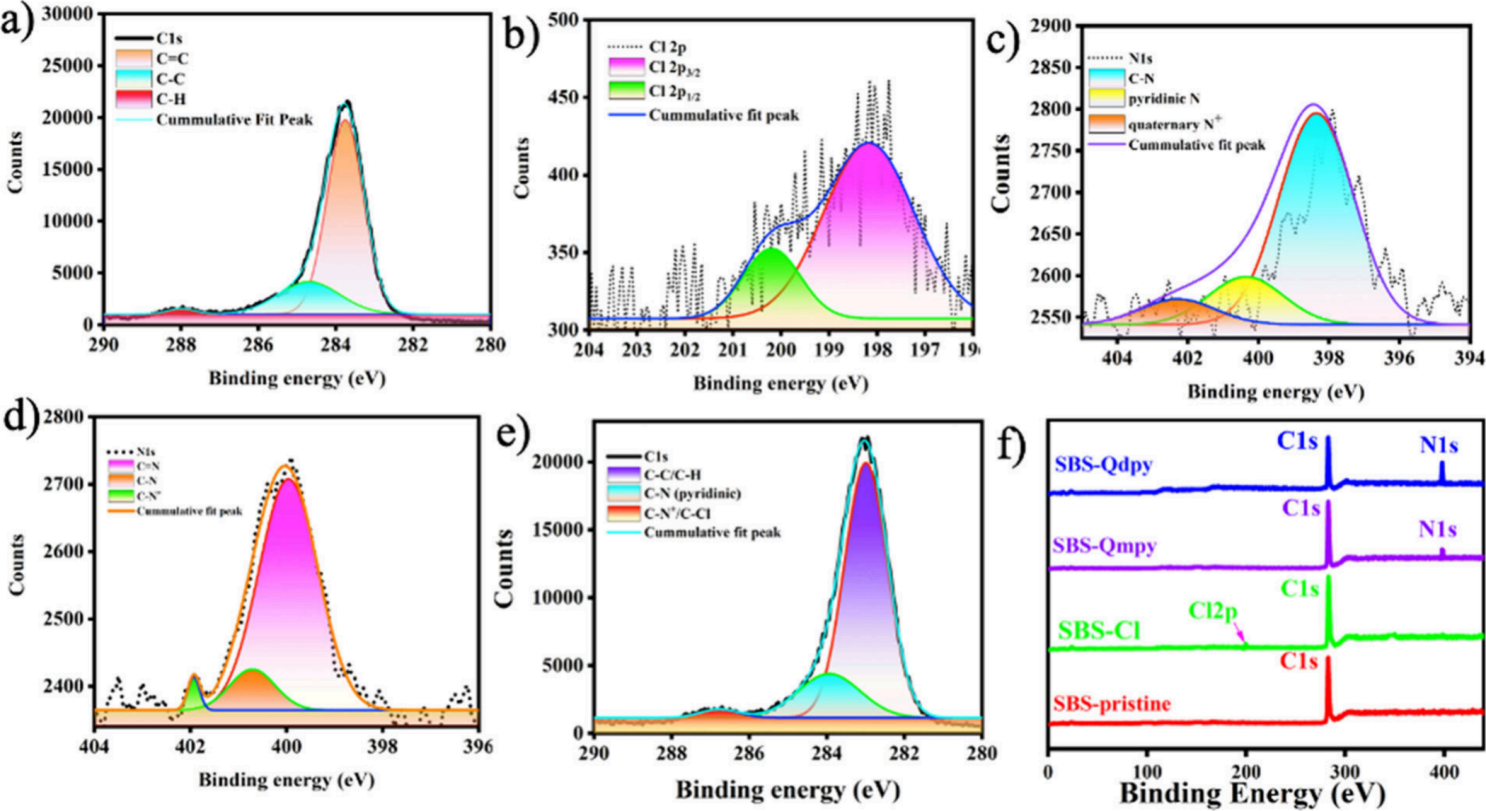
XPS spectra: (a) C 1s of SBS, (b) Cl 2p and (c) N 1s of SBS-dpy, (d) N 1s of SBS-mpy, (e) spectra of intermediates, and (f) wide-scan survey of SBS, CSBS, and SBS-QA^+^py.

### 3.2 Membrane Morphology and Computational Insights via DFT

As shown in Figures 3a–d, the surface and corresponding cross-sectional SEM images of SBS-QA^+^py AEMs reveal an intact, smooth, and void-free morphology, indicative of a uniform and densely packed microstructure. The effective incorporation of pyridine derivatives is evident because their presence preserves the microphase-separated morphology while facilitating the formation of continuous hydrophilic ionic domains due to their inherent basicity and electron density.^33^ Figure S4 further shows a transparent, light-yellow membrane surface with densely interconnected networks, reflecting a high mechanical integrity. Notably, the membranes exhibit remarkable flexibility, withstanding repeated bending and cribbling without visible cracks, attributed to the elastomeric nature of the SBS backbone and noncovalent stacking interactions between the pyridinic moieties and the PS segments of the base polymer.

**Figure 3:**
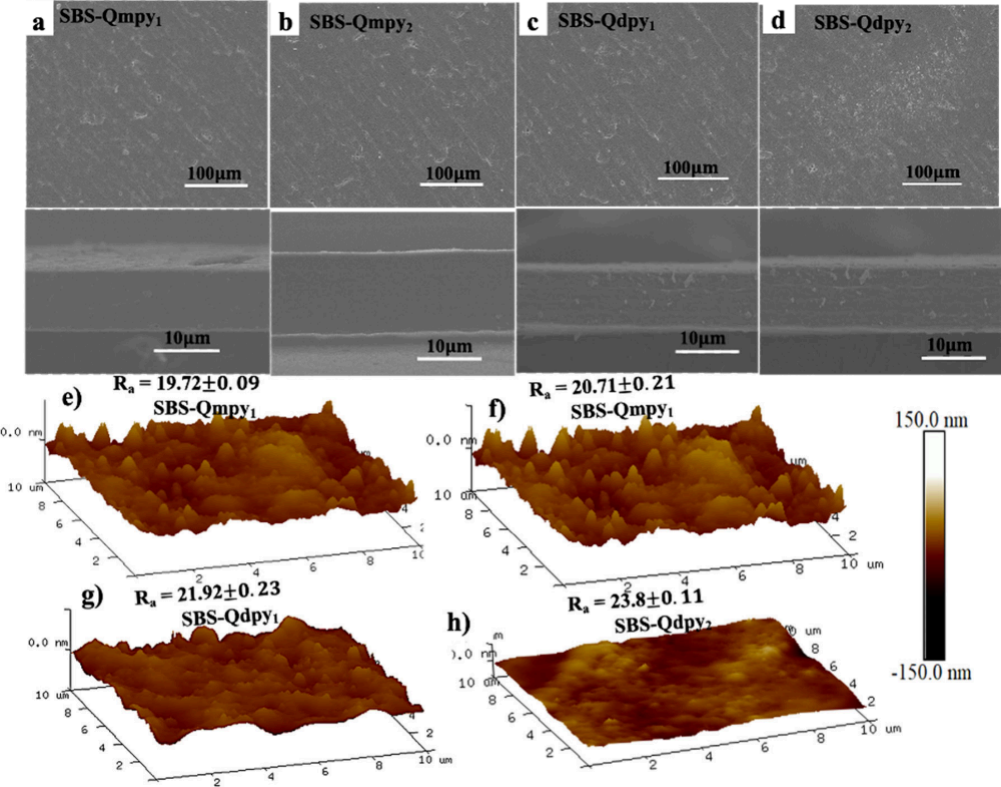
(a–d) Surface and corresponding cross-sectional SEM images and (e–h) AFM images.

Well-defined hydrophobic/hydrophilic phase separation in AEMs enhances water diffusion and facilitates the formation of efficient ion-transport channels.^34^ SBS-QA^+^py membranes exhibit clear microphase separation, promoting effective ion transport, as confirmed by AFM and SAXS analyses. In the AFM images (Figure 4e–h), dark regions represent the hydrophilic domains of pyridinium-functionalized segments, while bright regions correspond to the hydrophobic SBS backbone. Increasing the QA^+^py loading content ratios from 10% to 20% leads to greater surface roughness and enlarged hydrophilic domains, indicating more pronounced phase separation. In contrast, lower cationic loading results in smoother surfaces and clearer domain boundaries due to the uniform distribution of ionic moieties.

**Figure 4:**
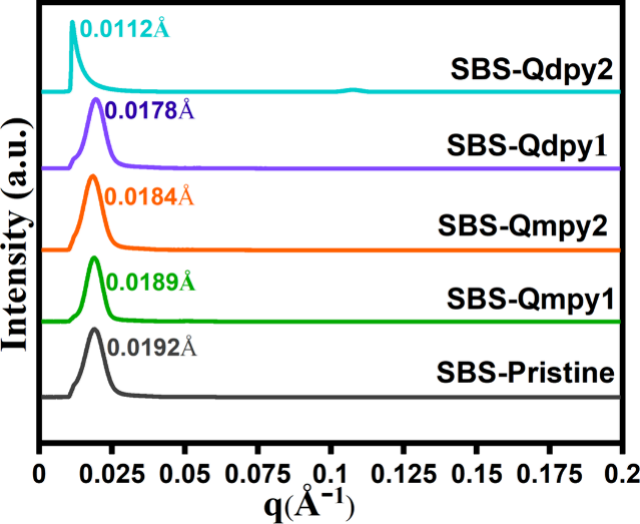
SAXS spectra of the SBS-QA^+^py AEMs.

SAXS was used to examine the nanoscale morphology and periodicity (*d* = 2π/*q*) of SBS membranes functionalized with hydrophilic pyridinium moieties, as shown in Figure 4, as a function of scattering vector *q*. It elucidates the structural evolution of SBS upon pyridinium derivative functionalization, revealing a systematic increase in the interdomain spacing from 33.23 nm (0.0189 Å^–1^) in SBS-Qmpy_1_ to 55.08 nm (0.0112 Å^–1^) in SBS-Qdpy_2_. Compared to pristine SBS (32.71 nm, 0.0192 Å^–1^), this expansion reflects the reorganization of ionic domains, driven by electrostatic interactions and hydration effects. The correlation between the *d*-spacing, IEC, and WU suggests that higher ion content amplifies electrostatic repulsion and osmotic swelling, intensifying phase separation. SBS-Qdpy_2_, exhibiting the largest *d* spacing, confirms this synergy, wherein pronounced hydrophilic domain segregation enhances ion transport.^35^ However, excessive swelling compromises the mechanical integrity, underscoring the need for a precise hydration–structural balance. SAXS and AFM reveal that ion clustering and nanoscale heterogeneity dictate the AEM performance, ensuring a balance between the conductivity and stability.

### 3.3 Physicochemical, Thermal, and Mechanical Properties of the Membranes

According to the Grotthuss mechanism, OH^–^ transport in AEMs requires adequate hydration, governed by WU,^36^ IEC, and SR. As shown in Figure 5a,b, WU and SR of SBS-QA^+^py membranes increase with temperature due to enhanced molecular ionic mobility and the higher density of hydrophilic pyridine derivatives, while dimensional swelling remains limited by π–π stacking between PS segments and pyridinic moieties. The WU and SR of SBS-QA^+^dpy AEMs were quantified based on water absorbed per unit dry mass and dimensional changes across temperature ranges. WU increased from 46.6 to 81.7%, and SR ranged from 28.3% to 34.1%, both correlating with the IEC at 80 °C, reflecting their elevated IECs (Table 1).

**Figure 5:**
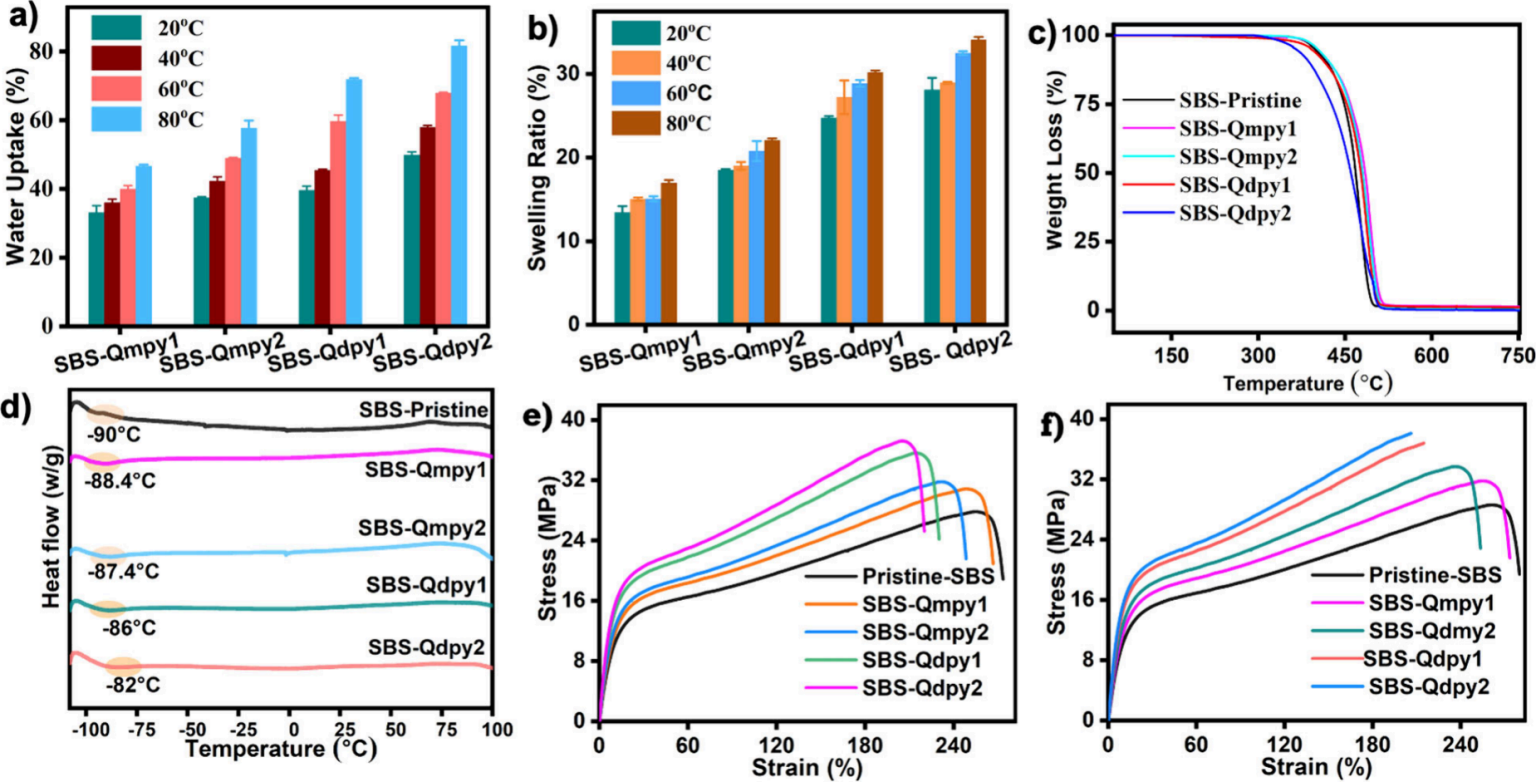
(a) WU, (b) SR, (c) thermal stability analysis, (d) glass transition analysis, and mechanical tensile properties of SBS-QA^+^py AEMs in OH^–^ form both under (e) dry and (f) wet states at room temperature.

**Table 1:** Membrane Properties of SBS-QA^+^py AEMs

	IEC (mequiv g^–1^)		WU (%)		SR (%)		λ^d^		
AEMs	experimental^a^	theoretical^b^	30 °C	80 °C	30 °C	80 °C	30 °C	80 °C	WCA (deg)^c^
SBS-Qmpy_1_	1.24 ± 0.91	1.25 ± 0.91	29.98 ± 2.02	46.61 ± 0.9	13.47 ± 0.73	28.31 ± 1.4	13.42	20.88	63.31
SBS-Qmpy_2_	1.47 ± 0.31	1.52 ± 0.31	35.02 ± 0.99	57.77 ± 0.23	15.41 ± 0.22	28.97 ± 0.10	13.47	21.83	54.08
SBS-Qdpy_1_	1.64 ± 0.74	1.67 ± 0.74	42.99 ± 0.01	71.87 ± 0.12	15.99 ± 0.31	32.52 ± 0.22	14.30	23.91	47.94
SBS-Qdpy_2_	1.72 ± 0.09	1.77 ± 0.09	44.62 ± 0.43	81.77 ± 1.54	16.99 ± 0.34	34.12 ± 0.34	14.874	26.41	42.15

a Hydration number as per WU and IEC.

b From a standard back-titration method.

c Calculated from ^1^H NMR spectra.

d Water contact angle at 25 °C.

The values range from the IEC used to quantify the concentration of cationic sites per unit mass of AEM, which dictates WU, hydration, and hydroxide conductivity.^37^ This parameter is determined experimentally by Mohr back-titration and theoretically based on the ^1^H NMR integrals of the functional groups. As shown in Table 1, the membranes exhibited IEC values of 1.24–1.72 mequiv g^–1^ experimentally and 1.25–1.77 mequiv g^–1^ theoretically. These results exhibit minor deviations attributable to measurement variability and variations in the polymer microstructure.^38^ Thus, WU and IEC are key metrics interlinked to optimize the ionic diffusion, mechanical stability, and membrane durability.

The hydration number (λ), another metric property of AEMs, quantifies the number of water molecules per quaternized pyridinium unit in AEMs, offering a precise measure of water organization and diffusivity within microphase-separated domains, which governs hydroxide transport and membrane durability.^39^ As summarized in Table 1, SBS-QA^+^py AEMs exhibit λ values ranging from 20.88 to 26.41 at 80 °C, as functions of WU and IEC. SBS-Qdpy_2_ attains the highest λ of 26.41, reflecting enhanced hydration shells around cationic sites. Membranes such as SBS-Qdpy_2_, SBS-Qmpy_2_, and SBS-Qmpy_1_ achieve the optimal λ that balances dimensional stability with ionic conduction, WU, and IEC. A properly tuned λ facilitates rapid hydroxide transport via the Grotthuss mechanism through well-connected water channels, while excessive hydration dilutes the cationic density, reducing conductivity through partial cation leaching.^40^ Pyridine-functionalized SBS AEMs combine ordered functional domains with a uniform water distribution to maintain high IEC and structural integrity. These findings underscore the critical interplay of hydration and IEC in the design of microstructural water networks for high-performance AEMFC membranes.

The wettability and hydrophilicity of the membranes were characterized by WCA measurements. As shown in Figure S6, the WCA of SBS-QA^+^py AEMs decreased with pyridinium loading, confirming improved wettability.^41^ This improvement arises from the incorporation of polar, electron-rich nitrogen groups that elevate the membrane’s surface polarity, facilitating strong ion–dipole interactions with water molecules. Higher quaternized cationic site densities disrupt the intrinsic microphase-separated morphology of SBS, promoting the formation of continuous hydrophilic domains. Consequently, this morphological transition reduces the surface energy and elevates water uptake. Specifically, pristine SBS showed a WCA of 77.9°, while SBS-Qmpy_1_ and SBS-Qmpy_2_ achieved 67° and 61.2°, with water uptakes of 44.97% and 51.01%, respectively. SBS-Qdpy_1_ and SBS-Qdpy_2_ displayed even lower WCAs of 43° and 41.2%, accompanied by water uptakes of 67.91% and 81.77% at 80 °C. These findings underscore the role of pyridine derivative loading in enhancing AEM hydrophilicity, which is critical for optimizing ion transport and water management in advanced electrochemical devices.

Mechanically robust and tough membranes are essential for high performance and long-term stability in AEMFCs. The tensile properties of SBS-QA^+^py membranes in the OH^–^ form were evaluated under dry and wet conditions at room temperature^42^ (Figure 5e,f). Pristine SBS exhibited high elongation (EB ≈ 258–264%) but moderate strength (TS ≈ 28–29 MPa), due to its rubbery PB and rigid styrene domains. Quaternization increased TS while slightly reducing EB. SBS-Qdpy_2_ showed the highest TS (37 MPa dry; 38 MPa wet) with moderate EB (206–215%), driven by ionic interactions and π–π stacking among aromatic domains. SBS-Qmpy membranes maintained a higher flexibility (EB ≈ 231–251% dry; 237–253% wet) with moderate TS (31–34 MPa). Hydration slightly lowered the TS and increased the EB through water-induced plasticization, demonstrating that ionic reinforcement combined with the elastomeric SBS backbone effectively balances strength and ductility, yielding mechanically resilient AEMs suitable for durable fuel cell operation.

The thermal stability of SBS-QA^+^py membranes was evaluated under a N_2_ atmosphere at a heating rate of 10 °C min^–1^ from 30 to 800 °C, a key factor in the AEM performance. As shown in Figure 5c, all SBS-QA^+^py membranes, including the pristine tripolymer, exhibited negligible weight loss below 100 °C, indicating the successful removal of residual solvents. The first major decomposition stage (100–460 °C) corresponds to the degradation of quaternized pyridinium groups. Among the membranes, SBS-Qdpy_2_ showed an earlier onset of degradation around 300 °C, likely due to its higher functional loading and increased water retention. The second decomposition stage occurred above 460 °C and is associated with degradation of the polymer backbone, with the mass loss around 440 °C reflecting the loss of quaternized groups within this stage. This decomposition temperature, well above 150 °C, exceeds the typical operating range of AEMFCs.^43^ All membranes showed slow degradation and backbone stability up to 500 °C, indicating strong interionic interactions and thermal robustness for the AEMFC operation. Furthermore, the phase transitions of AEMs were analyzed by employing differential scanning calorimetry to point out the thermal phase transition, thermal stability, and hydration behaviors of AEMs, which directly impact the mechanical stability and ion conductivities. Pristine SBS exhibits two distinct glass transition (*T*_g_) values: *T*_g1_ at a temperature of approximately −90 °C, attributed to the PB segments (70% of the tricopolymer), and *T*_g2_ in the vicinity of 100 °C, attributed to the PS segments (30% of the tricopolymer).^44^ The *T*_g_ values of the prepared membranes, such as SBS-Qmpy_1_, SBS-Qmpy_2_, SBS-Qdpy_1_, and SBS-Qdpy_2_, display phase-separated glass transitions compared to those of pristine SBS. This is due to the integration of pyridinium derivatives into the PB segments that enhance the hydrophilicity. As shown in Figure 5d, each of those AEMs exhibited different *T*_g_ values as a result of pyridinium derivatives that restricted the movement of the glassy states of the polymer backbone. Notably, the *T*_g_ of the PS block present in all copolymers did not affect the *T*_g_ values due to the smallest percentages in the polymer compositions. Moreover, the chlorination of PB segments in SBS, followed by quaternization, resulted in an increase in its *T*_g_ values to −82 °C (SBS-Qdpy_2_) compared to pristine SBS of −94 °C. This is due to the incorporation of pyridinium ionic clusters, which enhance the rigidity and polarity of the polymer by restricting the mobility of the glassy PB segments.^45^ Likewise, the *T*_g_ values of SBS-Qmy_1_, SBS-Qmpy_2_, and SBS-Qdpy_1_ AEMs run from −88 to −86 °C, which reveals the improvement of the hydrophilicity and the restrictions of the movements of the rubbery states of the PB segment.

### 3.4 Ionic Conductivity, Stability, and Computational Insights via DFT

Ionic conductivity is a key parameter in AEMs performance and directly measures ion-transport capability and its overall functionality; it depends on the IEC, chain mobility, and matrix structure. Furthermore, the dispersion of cationic moieties, surface functionalization, and compatibility between the polymer backbone and cationic moieties are crucial for enhanced ionic conductivity of AEMs.^46^ Figure 6a illustrates the temperature dependence of the hydroxide ion conductivity in various hybrid AEMs at 100% relative humidity. Despite consistent IEC values across all membranes, the hydroxide ion conductivity increases significantly with the cationic concentration and temperature. SBS-Qdpy_2_ achieves a maximum ionic conductivity of 101.23 mS cm^–1^ at an IEC of 1.72 mequiv g^–1^, while the chloride ion conductivity peaks at 51.23 mS cm^–1^ at 80 °C, as illustrated in Figure 6c. This disparity arises because hydroxide ions possess higher ionic conductivity than chloride ions due to their intrinsic smaller size, greater mobility, and efficient solvation tendencies, allowing them to traverse the membrane more swiftly than chloride ions.^47^ Furthermore, the ion conductivities of AEMs simultaneously rise with a rise of the IEC and WU. As mentioned earlier, the ionic conductivity of SBS-Qdpy_2_ AEM reached 101.23 mS cm^–1^ at 80 °C due to its high hydration efficiency, balanced WU, and uniform dispersion of pyridinium ions within the nanostructured matrix of the base polymer. Relatively, the SBS-Qmpy_1_ AEM exhibits the lowest ionic conductivity due to its low IEC and limited WU, which reduce rapid and efficient ion transport via the micropores of the membranes. However, higher WU can also degrade conductivity via dissolving the cationic moieties.

**Figure 6:**
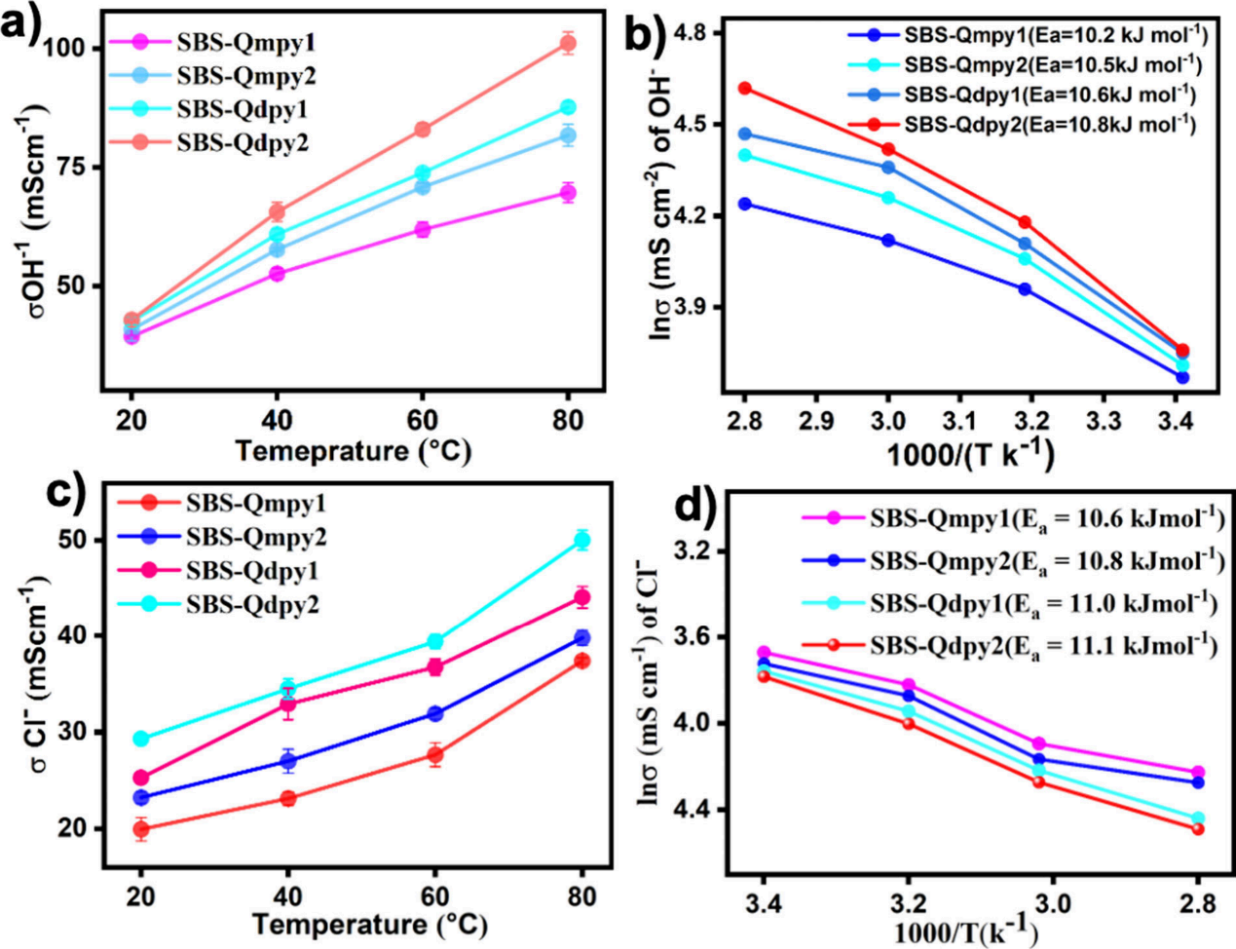
(a and b) Hydroxide conductivity and Arrhenius plots. (c and d) Chloride conductivity and Arrhenius plots of SBS-QA^+^py AEMs.

The activation energy (*E*_a_) offers insight into the ion-transport efficiency of the membrane. The SBS-Qdpy membrane exhibits a relatively low *E*_a_, which can be attributed to its flexible triblock architecture and nanostructured ionic domains formed by pyridinium functionalization, enabling efficient hydroxide ion conduction. As shown in Figure 6b, *E*_a_ values of the hydroxide conductivity of the membranes were determined from Arrhenius plots and ranged from 10.20 to 10.80 kJ mol^–1^ while the *E*_a_ value of chloride ions ranged from 10.60 to 11.08 kJ mol^–1^, which agrees with the Grotthuss mechanism of hydroxide conductivities, as illustrated in Figure 6d. As noted, SBS QA^+^py AEMs, with relatively low *E*_a_, demonstrate enhanced ion-transport efficiency and improved ionic conductivity.

**Figure 7:**
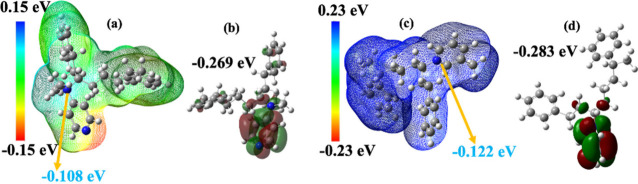
(a) Optimized electrostatic potential map of SBS-Qdpy. (b) LUMO of SBS-Qdpy. (c) Optimized electrostatic potential map of SBS-Qmpy. (d) LUMO of SBS-Qmpy.

AEMs are prone to degradation under alkaline conditions due to nucleophilic attacks by OH^–^ ions on QA groups and polymer backbones. Therefore, achieving sufficient alkaline stability is critical for their practical application.^48^ The oxidative stability of the SBS-QA^+^py AEMs were evaluated by simulating the attack of OH^–^ radicals using Fenton’s reagent at room temperature for 10 days. The results demonstrate that the membranes exhibit remarkable oxidative stability, retaining their structural integrity without visible cracking while maintaining a mechanical rigidity. As shown in Figure 8a, a slight weight loss was observed for the SBS-QA^+^py AEMs, which may be attributed to the partial loss of ionic residues and minor degradation of quaternary ammonium groups under the harsh oxidative environment induced by Fenton’s reagent. These findings underscore the robustness of the membranes and their potential durability under long-term operational conditions. This is due the fact that the prepared membranes were free from hydroxide attack on susceptible functional groups such as benzylic and ether linkages.

**Figure 8:**
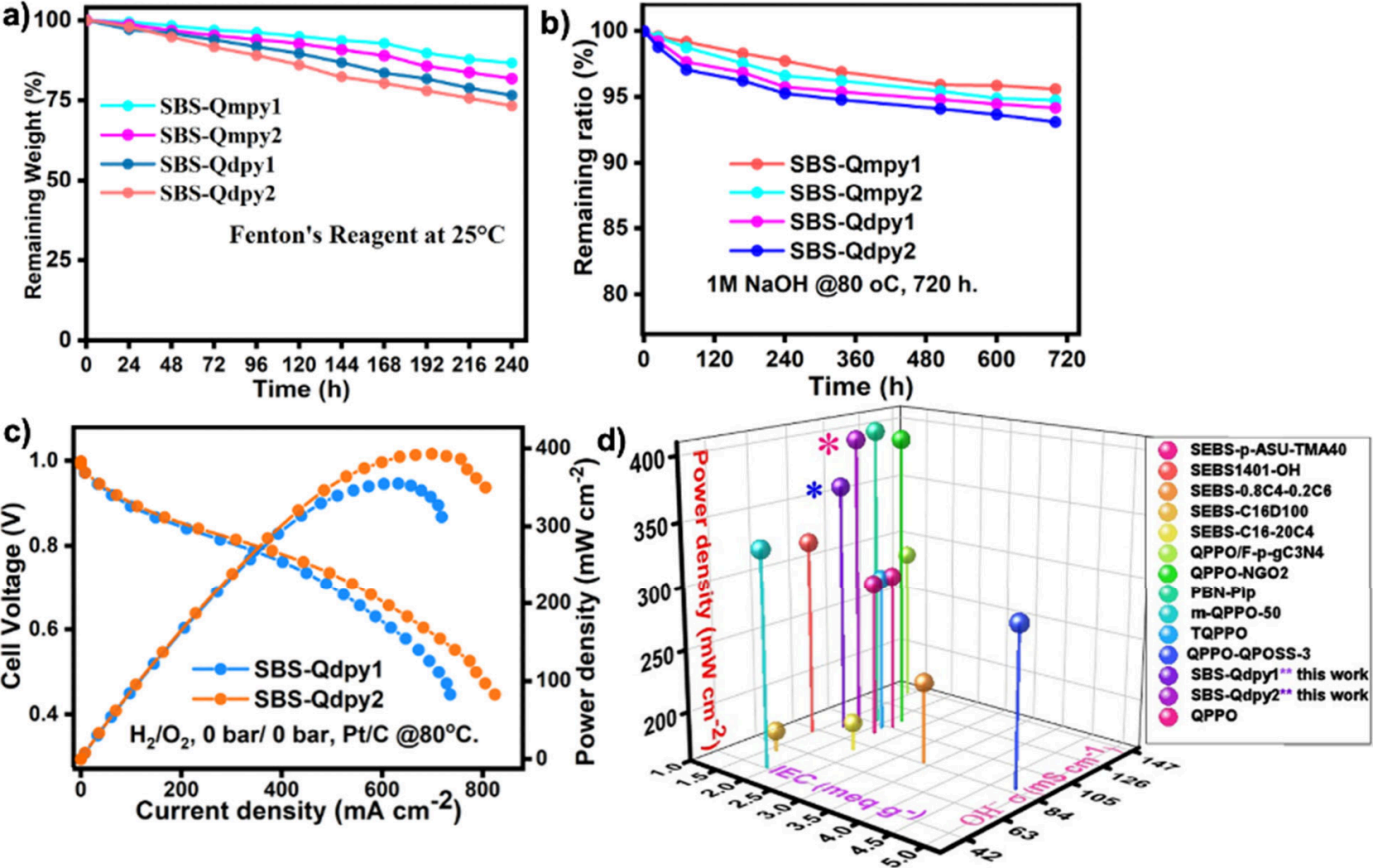
(a) Fenton’s oxidative stability. (b) Hydroxide stability. (c) H_2_/O_2_ fuel cell performance of SBS-Qdpy. (d) Comparison of SBS-QA^+^py AEMs with recent literatures, respectively.

The alkaline stability of the AEMs was evaluated by immersing the membranes in 1 M NaOH at 80 °C for 720 h, with IEC and hydroxide conductivity measurement every 72 h and the alkaline solutions refreshed periodically (Figure 8b). As summarized in Table 2, both parameters remained unchanged, indicating excellent alkaline stability of the SBS-QA^+^py AEMs. The chemical integrity of SBS-Qdpy was additionally examined by ^1^H NMR after alkaline treatment, and the spectrum (Figure 9d) showed no detectable structural changes, confirming its strong resistance to alkaline degradation.

**Table 2:** Durability Test of SBS-QA^+^py: IEC versus OH^–^ Conductivity before and after 720 h of Exposure in 1 M NaOH at 80 °C

	titration IEC (mequiv g^–1^)		OH^–^ σ (mS cm^–1^)	
AEMs	before	after	before	after
SBS-Qmpy_1_	1.24 ± 0.91	1.24 ± 0.23	69.72 ± 1.01 (80 °C)	68.92 ± 1.13 (80 °C)
SBS-Qmpy_2_	1.47 ± 0.31	1.46 ± 1.01	81.81 ± 2.31 (80 °C)	79.73 ± 1.34(80 °C)
SBS-Qdpy_1_	1.67 ± 0.74	1.64 ± 0.65	87.75 ± 1.14 (80 °C)	85.89 ± 0.24 (80 °C)
SBS-Qdpy_2_	1.72 ± 0.09	1.71 ± 0.43	101.23 ± 2.35 (80 °C)	98.93 ± 1.22 (80 °C)

**Figure 9:**
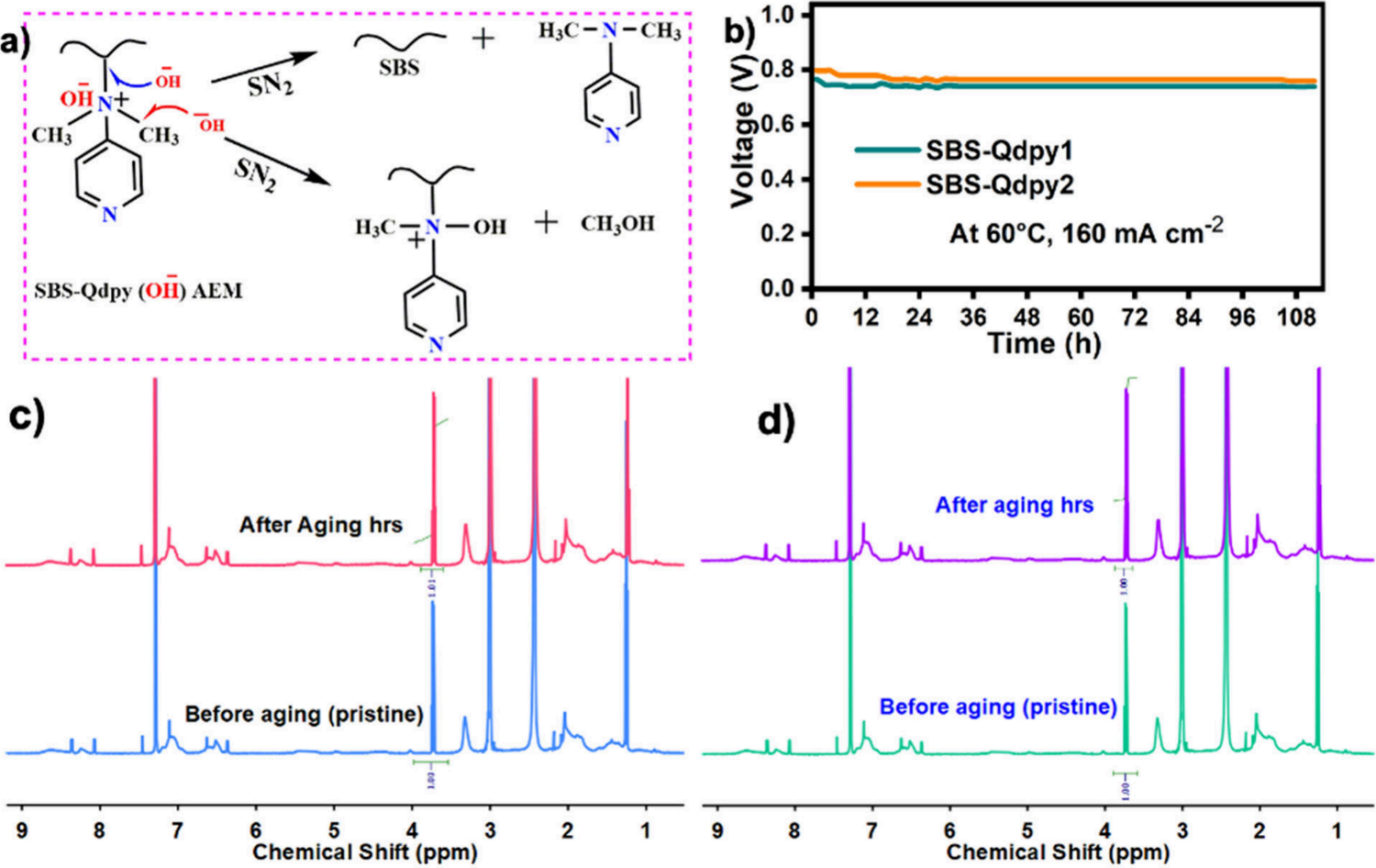
(a) Proposed degradation mechanism under long-term AEM operation. (b) Durability test of SBS-Qdpy at 160 mA cm^–2^. (c) ^1^H NMR spectrum of aged SBS-Qdpy after long-term operation. (d) ^1^H NMR spectrum of SBS-Qdpy after alkaline durability testing.

To probe the structural stability of the functionalized membranes, DFT calculations were conducted with a focus on LUMO energies. As shown in Figure 7b,d, SBS-Qdpy and SBS-Qmpy exhibited low LUMO values of −0.269 and −0.283 eV, respectively, suggesting enhanced geometric stability and resistance to alkaline degradation. This stability is attributed to electron cloud conjugation between PS and pyridine moieties, which promotes orbital delocalization, lowers the LUMO energy, and reduces the susceptibility to hydroxide attack, consistent with experimental alkaline stability results. ESP values further support this trend, with SBS-Qmpy (0.122 eV) and SBS-Qdpy (0.108 eV), as shown in Figure 7a,c, showing reduced susceptibility to OH^–^ attack, reinforcing their alkaline stability. This finding aligns with the results of the alkaline stability test. Furthermore, contour plots (Figure S5d,i) visualize electron density distributions, mapping interactions between cations and hydroxide ions within the polymer matrix.

### 3.5 Fuel Cell Performance and Durability

To evaluate the practical potential of the developed membranes, SBS-Qdpy_1_ and SBS-Qdpy_2_, identified as the most promising candidates in the SBS-QA^+^py series due to their balanced IEC and mechanical integrity, were tested in single-cell alkaline fuel cells. At 80 °C under fully humidified H_2_/O_2_ (100% RH) and ambient pressure, the membranes delivered peak power densities of 362.2 and 398.1 mW cm^–2^, respectively, surpassing those of the commercial Sustainion X37-50T (347 mW cm^–2^; Figure S8) under identical conditions and the PiperION-A20 membrane (377 and 355 mW cm^–2^)^56^ measured under comparable conditions except for backpressure. This enhanced performance reflects improved interfacial compatibility and efficient ion transport within the SBS-Qdpy architectures. The observed cell performance is also comparable to advanced SBS-derived and other functionalized AEMs (Figure 8d)^36,49,51,52,55,57–63^ and consistent with their robust physicochemical profiles (Table 3). These results confirm that the molecular design of the SBS-Qdpy series promotes interconnected ion-conduction channels, while maintaining structural resilience.

**Table 3:** Comparisons of Key Properties of SBS-QA^+^py with Benchmarked AEMs

AEMs	WU (%)	IEC (mequiv g^–1^)	OH^–1^ σ (mS cm^–1^)	stability conditions	ref
SEBS1401-OH	17.6%@30 °C	1.48	80.5 (80 °C)	2 M NaOH, 480 h, 60 °C (30%)	^49^
SBS-AA25	50.1 ± 1.2@20 °C	1.72	30.87 (80 °C)	2 M NaOH, 240 h, 80 °C (5.8%)	^10^
h-SBS-TMA1.83	139 ± 10@25 °C	1.83	93(80 °C)	1 M NaOH, 720 h, 80 °C (−%)	^50^
SEBS-P-ASU-TMA_40_	66.2@80 °C	2.16	96.6(80 °C)	2 M NaOH, 1000 h, 80 °C (17%)	^36^
SEBS-0.8C_4_-0.2C6	41.84@80 °C	3.42	85.27(80 °C)	2 M NaOH, 1400 h, 80 °C (5.76%)	^51^
SEBS-C_16_D_100_	82.2 ± 1.3@20 °C	1.59	57.5	2 M NaOH, 1300 h, 80 °C	^52^
HQA-F_5_-SEBS	136.7 ± 10.2@80 °C	1.57	87.0 ± 3.2	1 M KOH, 500 h, 80 °C (−%)	^53^
X-e SBS_25_-TMA-3.01	79.0@30 °C	3.01	153	1 M NaOH, 500 h, 80 °C (97%)	^54^
SEBS-C16-20C4		2.35	77.78	1 M NaOH, 500 h, 80 °C (94.13%)	^55^
SBS-Qdpy_1_	87.87 ± 0.123@80 °C	1.67	87.75 ± 1.14 (80 °C)	1 M NaOH, 720 h, 80 °C (−%)	this work
SBS-Qdpy_2_	101.23 ± 1.54@80 °C	1.72	101.23 ± 2.35 (80 °C)	1 M NaOH, 720 h, 80 °C (−%)	this work

Durability testing at a constant current density of 160 mA cm^–2^ (Figure 9b) demonstrated stable voltage retention over 112 h, and post-test ^1^H NMR analysis (Figure 9c) revealed minimal structural changes. This operational stability indicates a strong resistance to cationic degradation and mechanical stress, essential for long-term AEMFC operation. Although the membranes’ power output approaches that of next-generation AEMs, the optimized synergy of IEC, dimensional stability, and mechanical strength ensures superior durability and scalable fabrication. Remaining performance gaps relative to benchmark systems could be addressed through improvements in humidity management, catalyst–ionomer interfaces, and MEA engineering, highlighting clear avenues for further advancement.

## 4 Conclusion

Here, we report a new class of pyridinium-functionalized SBS AEMs synthesized through controlled chlorination of the elastomeric PB segment and direct solution-casting quaternization methods. The resulting SBS-QA^+^py series of membranes exhibit a balanced hydrophobic–hydrophilic architecture, yielding restricted segmental mobility (*T*_g_ ≈ −82 °C), strong noncovalent π–π interactions, and well-defined ion-conducting domains. These structural features collectively enable high mechanical resilience (38 MPa, 237% elongation in the wet state; 37 MPa, 251% elongation in the dry state), excellent hydration and water retention (WU = 81% at 80 °C), and robust dimensional stability (≈34% at 80 °C). The membranes deliver competitive IEC values (1.42–1.72 mequiv g^–1^) and efficient long-range hydroxide transport, achieving ionic conductivities up to 101 mS cm^–1^ at 80 °C. Their alkaline durability and mechanical integrity support peak power densities of 362–398 mW cm^–2^ in H_2_/O_2_ fuel cells, with stable operation over 100 h without detectable loss of voltage. Overall, the triblock SBS-QA^+^py AEMs design provides a versatile and scalable platform for high-performance AEMs, where phase-segregated morphology, reinforced ion clustering, and elastomeric toughness work synergistically. Further refinement of ionic group stability and tunable cross-linking is expected to unlock even higher performance in next-generation alkaline fuel cell systems.

## Supplementary Material



## Data Availability

All data supporting this study are available upon request.

## Notes

Chlorination reagent ratios and calculations (Table S1), chlorination reaction conditions and ratios (Table S2), quaternization ratios of CSBS with pyridine derivatives (Table S3), ^1^H NMR spectra of pyridine derivatives (Figure S1), FT-IR spectra of pristine SBS, CSBS, and SBS-QA^+^py AEMs (Figure S2), GPC traces of SBS-QA^+^py AEMs and pristine SBS (Figure S3), digital photographs of SBS-QA^+^py AEMs (Figure S4), DFT-optimized geometries and electronic properties of SBS-Qmpy and SBS-Qdpy AEMs (Figure S5), WCAs of SBS-QA^+^py AEMs (Figure S6), proposed alkaline degradation pathway and ^1^H NMR spectrum of SBS-QA^+^py AEMs after alkaline treatment (Figure S7), AEMFC working principles and the ion flow channels (Figure S8), peak power density of commercially available Sustainion X37-50T AEMs (Figure S9), and peak power density of SBS-Qdpy_1_ and SBS-Qdpy_2_ AEMs at 60 °C (Figure S10) (PDF)
