# Supplementary material for: Enhanced Fuel Cell Performance with Robust Pyridinium-Derivative-Functionalized SBS Triblock Copolymer Anion-Exchange Membranes
Source: ACS Appl Mater Interfaces. 2026 Jan 20;18(4):6687–700. doi: 10.1021/acsami.5c06877 (PMC12884473; doi:10.1021/acsami.5c06877)
Supplement: Supplementary file 1 [file am5c06877_si_001.pdf]

Supporting information

Enhanced Fuel Cell Performance with Robust Pyridinium derivative-Functionalized SBS triblock Copolymer Anion Exchange Membranes

Beyadgalem Endawoke Anley[a], Yohannis Wondwosen Ahmed[a], Afandi Yusuf[c], Andy Candra[a], Sintayehu Leshe Kitaw[a], Tsung-Yun Wu[a], Chun-Chiang Huang[d], Jun-Sheng Wang[d], Darieo Thankachan[a], Mahvash Hira Khan[a], Yu-Ting Cheng[a], Chen-Hao Wang[c], Hsieh-Chih Tsai[a,b,e*]

a. Graduate Institutes of applied science and Technology, National Taiwan University of science and Technology, Taipei 106, Taiwan, ROC.
b. Advanced Membrane materials center, National Taiwan University of science and Technology, Taipei 106, Taiwan
c. Department of material science and Engineering, National Taiwan University of science and Technology, Taipei 106, Taiwan.
d. Taiwan Instrument Research Institute, National Applied Research Laboratories, Hsinchu 302, Taiwan.
e. R&D Center for Membrane Technology, Chung Yuan Christian University, Chungli, Taoyuan 320, Taiwan

Correspondence: h.c.tsai@mail.ntust.edu.tw (H.C.T) tel.: +886 -2-227303779 (H. C. T)

The synthesis conditions in Table (S1-S3) summarize a batch-to-batch study optimizing SBS chlorination. All reactions were conducted at 65 °C for 72 h with ~0.1 wt% AIBN, while polymer and solvent loadings were varied to assess scalability. Despite these variations, the chlorinated SBS products maintained consistent appearance, solubility, and reactivity. These insights guided the finalized synthesis for membrane fabrication, yielding intermediates with uniform reactivity toward pyridine derivatives and enabling membranes with stable fundamental properties.

Table S1. Chlorination Reagent Calculations for 1 g SBS.

**Batch**	**SBS (g)**	**$CHCl_3$ Ratio**	**$CHCl_3$ (g)**	**AIBN (% w/w)**	**AIBN (g)**
**1**	1.0	1:2	2.0	0.1%	0.001
**2**	1.0	1:3	3.0	0.5%	0.005
**3**	1.0	1:1	1.0	0.1%	0.001

Table S2. Chlorination Reaction Conditions.

**Batch ID**	**SBS (g)**	**$CHCl_3$ (g)**	**AIBN (g)**	**Toluene (mL)**	**Temp (°C)**	**Time (h)**	**BHT**	**Observation**
**C1**	1.0	2.0	0.001	40	60	72	Yes	Transparent, low viscosity
**C2**	1.0	3.0	0.005	40	60	72	Yes	Slightly viscous
**C3**	1.0	1.0	0.001	40	60	72	Yes	Thin liquid, good color

Table S3. Quaternization of Chlorinated SBS film with Pyridine Derivatives.

**Batch ID**	**Chlorinated SBS**	**Pyridine Type**	**Mol% Added**	**Solvent**	**Temp (°C)**	**Time (h)**	**Observation**
Qmpy10	C3	3-Methylpyridine (mpy)	10%	Toluene	40	48	Yellowish transparent film
**Qdpy10**	C4	4-Dimethylaminopyridine (dpy)	10%	Toluene	40	48	Light yellow transparent film
**Qmpy20**	C3	3-Methylpyridine (mpy)	20%	Toluene	40	48	Yellowish, transparent film
**Qdpy20**	C4	4-Dimethylaminopyridine (dpy)	20%	Toluene	40	48	Light yellow transparent film

Note: the ratios are calculated between polymer: chloroform: AIBN.

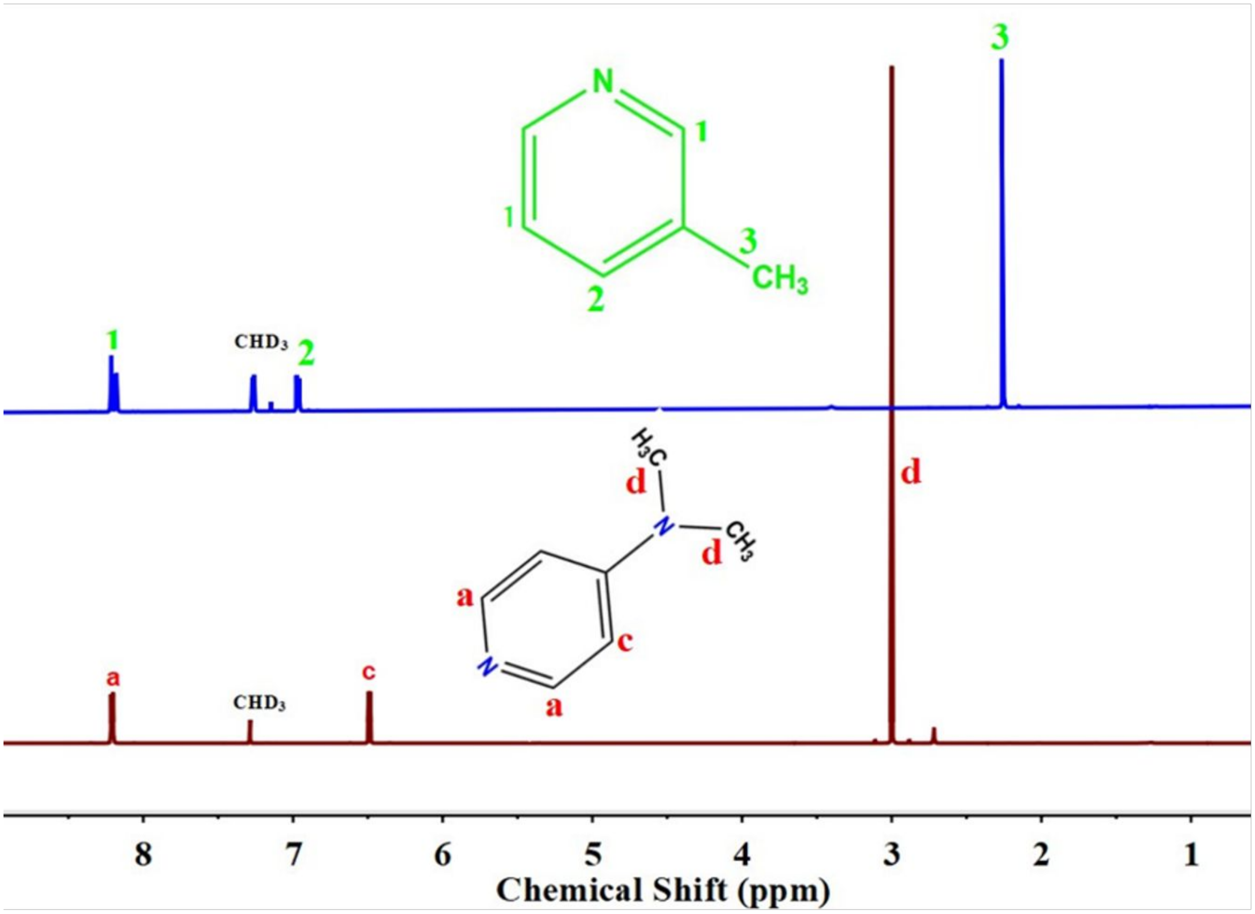

Figure S1: 1H NMR for pyridine derivatives;3-methyl pyridine (green, blue spectra) and 4-dimethylaminopyridine (black, brown spectra).

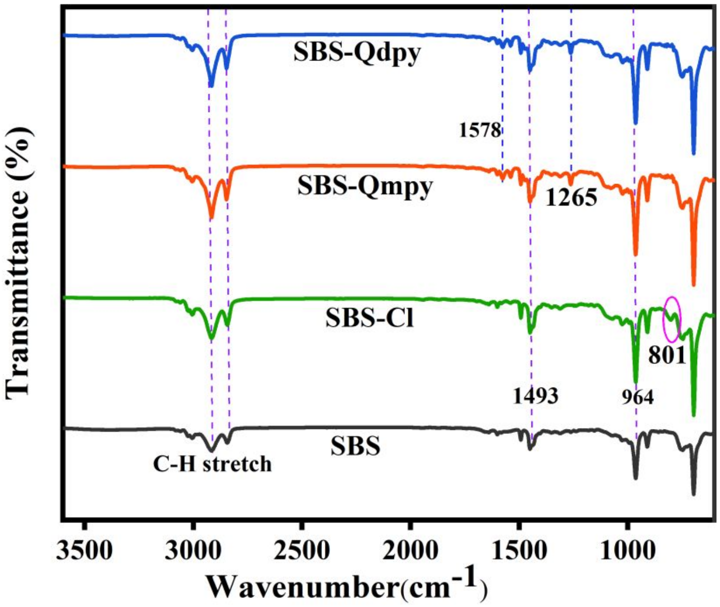

Figure S2: *ATR-FTIR spectra of pristine SBS, CSBS and SBS-QA+py AEMs*

The distinctive peaks at 1601 cm^{-1}, 1493 cm^{-1}, 1452 cm^{-1}, 964 cm^{-1}, and 696 cm^{-1} are attributed to the prominent vibrational modes of the polystyrene domains in the pristine SBS matrix, While the C-H stretching peaks at 2922 cm^{-1} and 2842 cm^{-1}, along with the vibrational peaks at 906 cm^{-1} and 756 cm^{-1}, correspond to the polybutadiene segment of SBS[1]. New peaks appear at 801 cm^{-1} due to the integration of C-Cl absorption in CSBS, resulting from the free radical chlorination of vinyl protons in SBS. The peaks at 1265 cm^{-1} and 1594 cm^{-1} arise from the quaternization of pyridinium derivatives. The disappearance of the peak at 801 cm^{-1} confirms the successful quaternization of the chlorinated tri-copolymer.

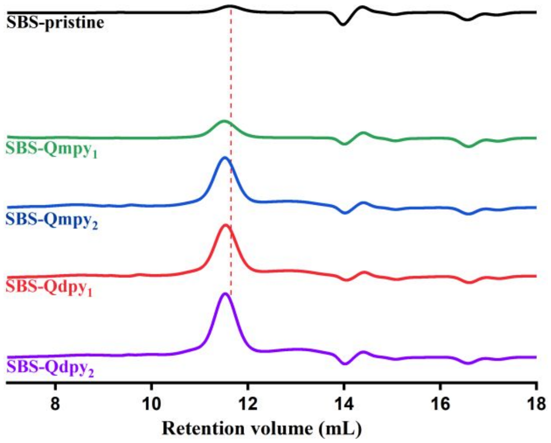

Fugure S3. GPC traces of SBS-QA^+py AEMs and the pristine SBS

The molecular weight distribution of SBS-QA^+py AEMs was determined using GPC employing Dimethylacetamide (DMC) as the solvent and monodisperse polystyrene as the internal standard. The pristine SBS exhibited a number-average molecular weight (Mn) of 140 kDa, with a corresponding polydispersity index (PDI) of 1.05. Upon integration with pyridinium derivatives, the PDI increased consistently, indicating a broader distribution of molecular weights, which reflects the modifications in the polymer structure as shown figure S2. As the molar ratio of

pyridine derivatives increased, the relative PDI indices (Mw/Mn) slightly rose consistently, ranging from 1.137 to 1.141, confirming uniform dispersion of pyridinium derivative (cationic) moieties and a stable, nearly uniform weight distribution within the polymer matrix.

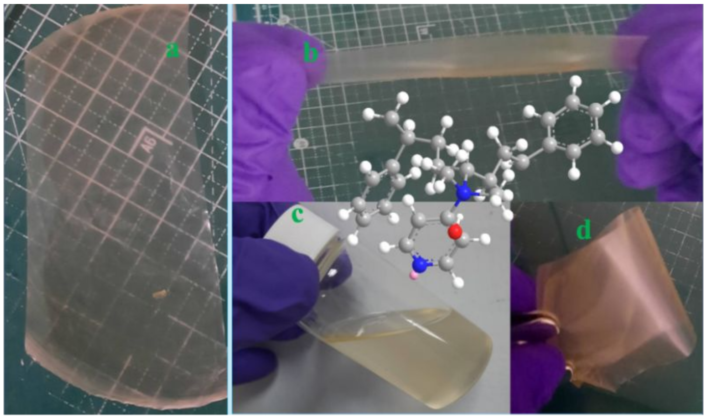

Figure S4: The digital physical photo of (a) SBS-QA$^+$py AEMS film, (b) stretched film, (c) solution for casting, and (d) folded image.

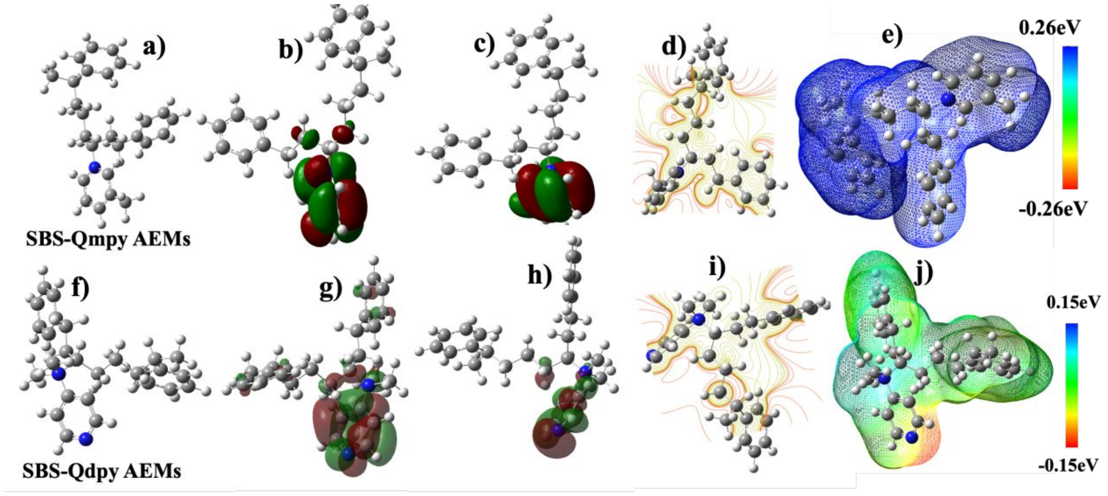

Figure S5: DFT-Optimized geometries and electronic properties of SBS-Qmpy and SBS-Qdpy AEMs, (a) SBS-Qmpy geometry, (b) LUMO, (c) HOMO, (d) Contourlines, (e) ESP, (f) SBS-Qdpy geometry, (g) LUMO, (j) HOMO, (i) Contourlines, (j) ESP.

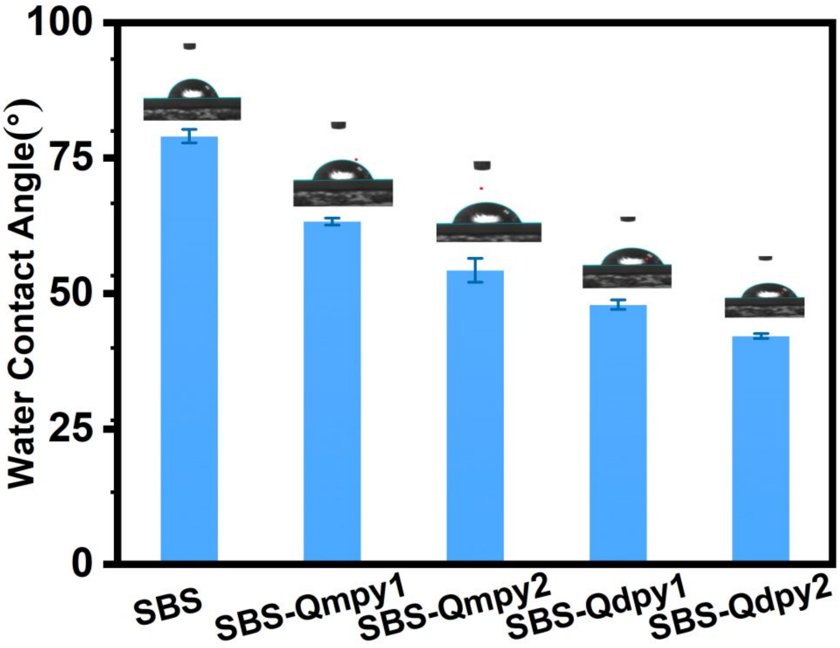

Figure S6: Water contact angle of SBS-QA$^+$py AEMs.

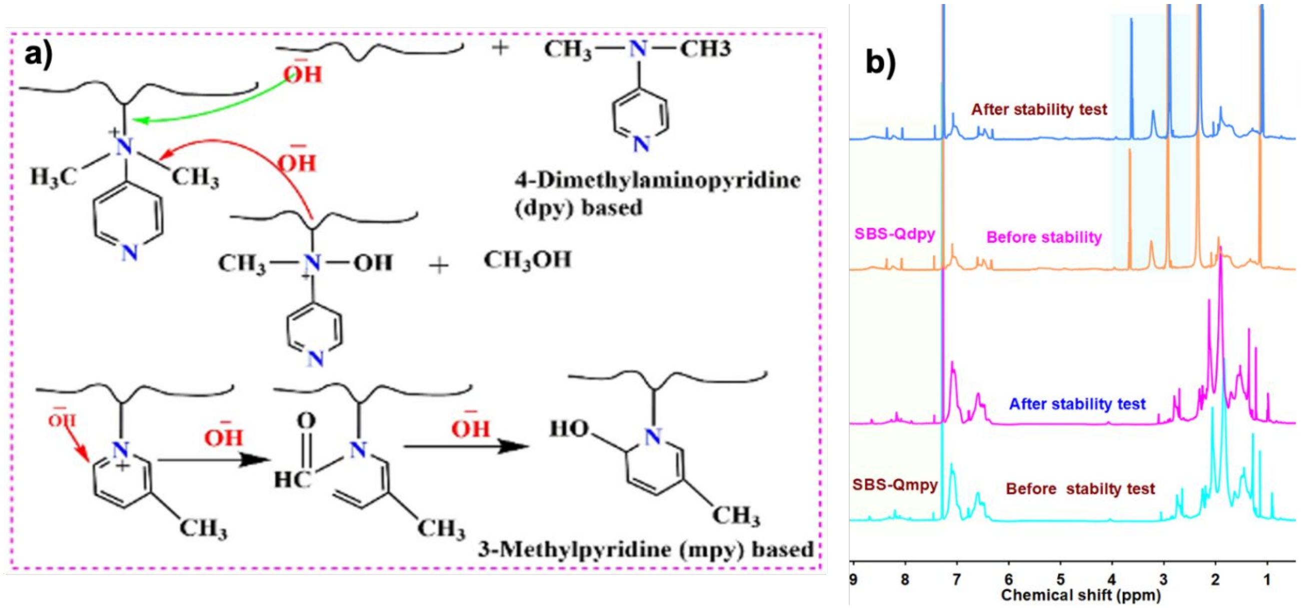

Figure S7. (a) Proposed degradation pathway schematic for the SBS-QA$^+$py AEMs under alkaline conditions. (b) ^{1}H NMR spectrum of the aged SBS-QA$^+$py AEMs after alkaline treatment.

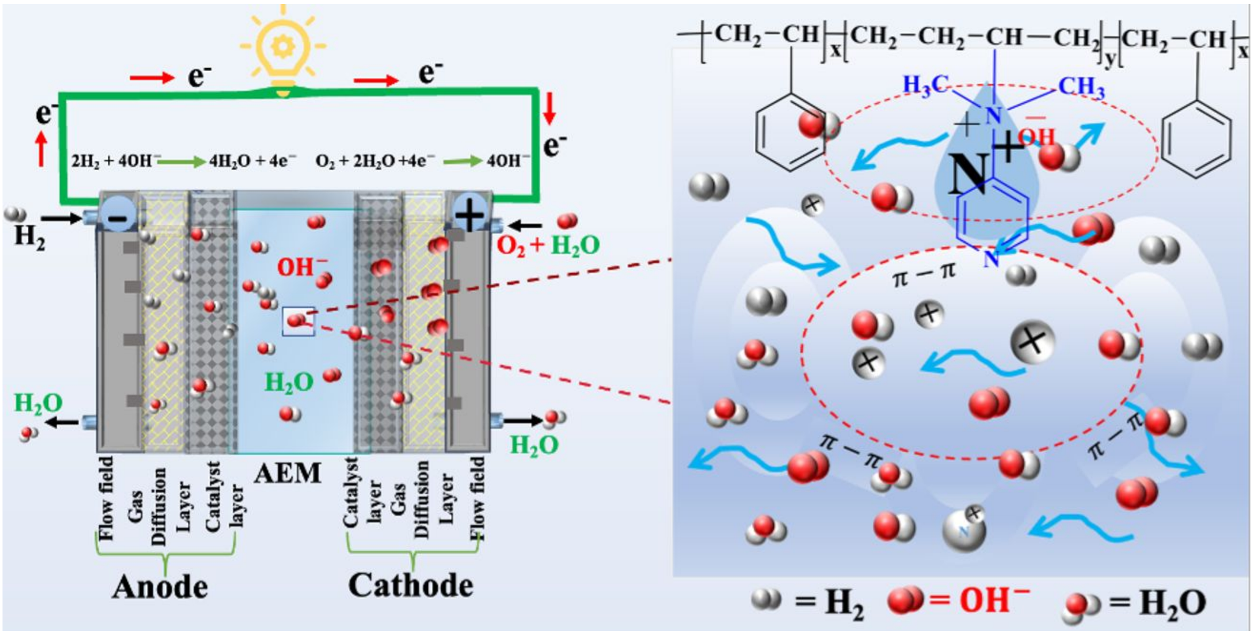

Figure S8: AEMFC working principles and the ion flow channels.

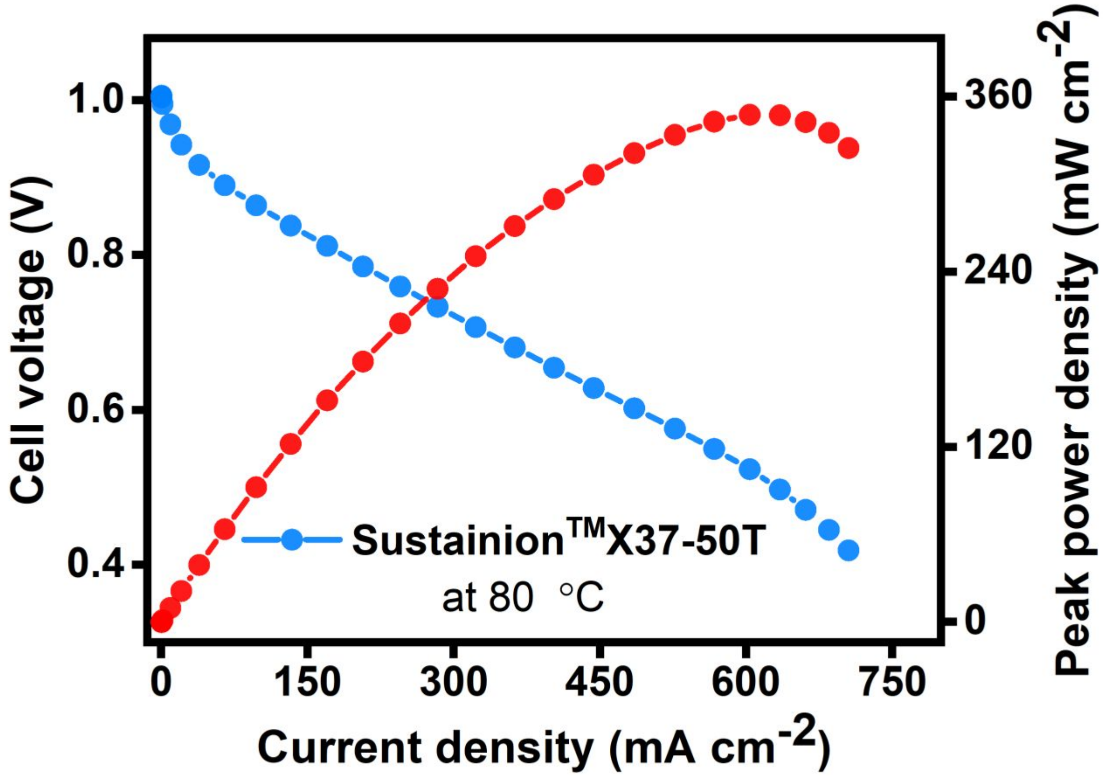

Figure S9: Peak power density of Comercially available Sustainion^{TM} X37-50T AEMs at 80℃ and 0 bar back pressure.

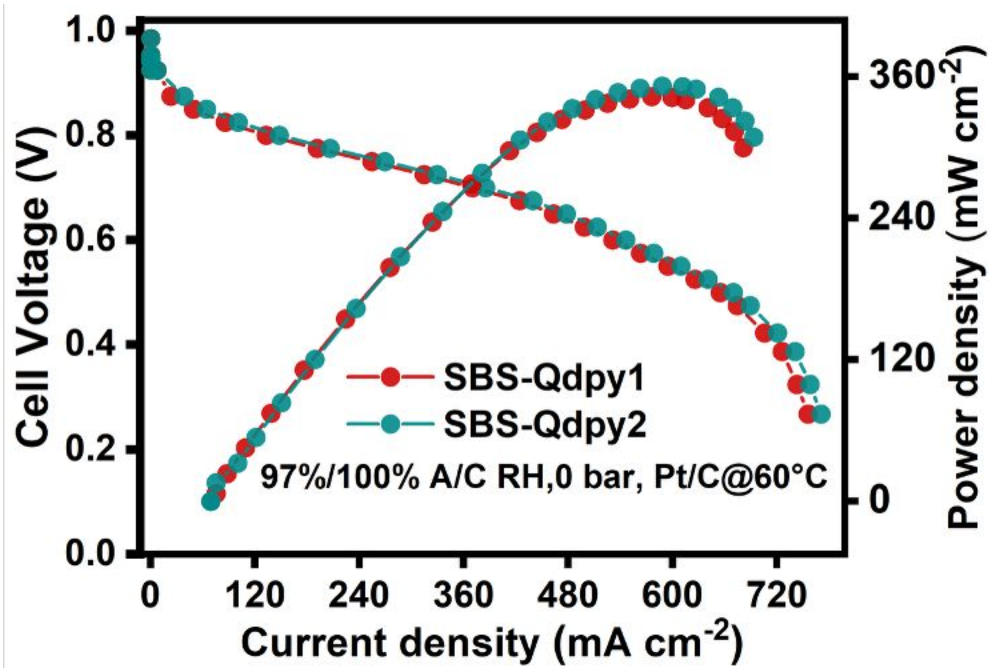

Figure S10:Peak power density of SBS-Qdpy1 and SBS-Qdpy2 AEMs at 60 ℃.

Reference

1. Gündüz, S.; Sar, Y.; Çaktı, K., EFFECT OF POLYBUTADIENE COMPOSITION ON THE GLASS TRANSITION TEMPERATURE OF SBS BLOCK COPOLYMERS. *Eskişehir Technical University Journal of Science and Technology A-Applied Sciences and Engineering* **2021,** *22* (1), 45-54.
